# Diffusiophoresis of Macromolecules within the Framework of Multicomponent Diffusion

**DOI:** 10.3390/molecules29061367

**Published:** 2024-03-19

**Authors:** Onofrio Annunziata

**Affiliations:** Department of Chemistry and Biochemistry, Texas Christian University, 2950 W. Bowie St., Sid Richardson Bldg. #438, Fort Worth, TX 76109, USA; o.annunziata@tcu.edu

**Keywords:** preferential hydration, Onsager reciprocal relations, Donnan equilibrium, Nernst–Planck equations, electrophoresis, polyethylene glycol, lysozyme

## Abstract

Diffusiophoresis is the isothermal migration of a colloidal particle through a liquid caused by a cosolute concentration gradient. Although diffusiophoresis was originally introduced using hydrodynamics, it can also be described by employing the framework of multicomponent diffusion. This not only enables the extraction of diffusiophoresis coefficients from measured multicomponent-diffusion coefficients but also their theoretical interpretation using fundamental thermodynamic and transport parameters. This review discusses the connection of diffusiophoresis with the 2 × 2 diffusion-coefficient matrix of ternary liquid mixtures. Specifically, diffusiophoresis is linked to the cross-term diffusion coefficient characterizing diffusion of colloidal particles due to cosolute concentration gradient. The other cross-term, which describes cosolute diffusion due to the concentration gradient of colloidal particles, is denoted as osmotic diffusion. Representative experimental results on diffusiophoresis and osmotic diffusion for polyethylene glycol and lysozyme in the presence of aqueous salts and osmolytes are described. These data were extracted from ternary diffusion coefficients measured using precision Rayleigh interferometry at 25 °C. The preferential-hydration and electrophoretic mechanisms responsible for diffusiophoresis are examined. The connection of diffusiophoresis and osmotic diffusion to preferential-interaction coefficients, Onsager reciprocal relations, Donnan equilibrium and Nernst–Planck equations are also discussed.

## 1. Introduction

A wide range of colloidal particles, such as inorganic nanoparticles, proteins, synthetic polymers and micelles, are subject to diffusion-based transport in complex liquids in a variety of biochemical, biotechnological, geochemical and industrial processes. These include centrifugation [1], dialysis [2], adsorption [3], crystallization [4], transport in microfluidics [5], living systems [6], gel media [7], insertion into dead-end pores [8], controlled release [9], reaction kinetics and pattern formation [10,11]. Since concentration gradients of colloidal particles and related additives such as salts, osmolytes and buffer components are normally encountered in these mass-transfer processes, diffusion coefficients play a crucial role in modeling the kinetic evolution of spatial concentration profiles of mixture constituents.

**Figure 1:**
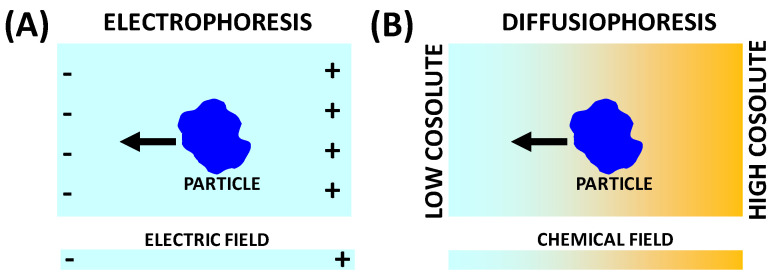
(**A**) Electrophoresis is particle migration in response to an electric field. If a particle is positively charged, migration occurs from high (+) to low (−) electric potential. (**B**) Diffusiophoresis (right) is particle migration caused by a gradient of cosolute chemical potential (chemical field). This is established by a concentration gradient of cosolute. The colloidal particle is explicitly shown, while cosolute and solvent molecules are described as a uniform background for simplicity, with the color gradient representing the cosolute gradient.

In recent years, a mass transport process known as diffusiophoresis has attracted considerable attention [12,13,14]. This is the net diffusive migration of a colloidal particle through a liquid induced by a directional concentration gradient of a cosolute at constant temperature and pressure. Although diffusiophoresis may occur in any liquid, this transport phenomenon is typically considered in the context of aqueous mixtures, in which the cosolute responsible for the migration of a water-soluble colloidal particle is a salt [14] or even a nonionic additive [15] such as an osmolyte [15,16]. As illustrated in Figure 1, diffusiophoresis is analogous to the well-known electrophoresis [15,17] of charged particles, which is caused by an external electric field, i.e., a gradient of electric potential. Correspondingly, diffusiophoresis is driven by a gradient of cosolute chemical potential. In other words, we can say that the diffusiophoresis of a colloidal particle is driven by the “chemical” field generated by the concentration gradients of a salt or osmolyte in water. Although most studies have focused on diffusiophoresis of charged colloidal particles in the presence of salt gradients [18,19,20,21,22], it is important to note that diffusiophoresis can also be observed for neutral colloidal particles [23,24,25]. Furthermore, the underlying mechanism responsible for diffusiophoresis is not unique as in the case of electrophoresis (electric field), but it can be varied by changing the chemical nature of the cosolute. This increases the complexity of diffusiophoresis as more than one mechanism can concur with the overall diffusiophoresis experienced by a colloidal particle. In this paper, the main mechanisms responsible for particle diffusiophoresis of macromolecules will be discussed.

Diffusiophoresis of a colloidal particle is characterized by the diffusiophoretic mobility, *D*_PS_, which is typically introduced by employing the following linear relation [13,19]:

(1) $$v_{\mathrm{PS}}=-\;\;D_{\mathrm{PS}}\;\nabla \;\ln C_{S}$$

where *v*_PS_ is particle diffusiophoretic velocity, and *C*_S_ is cosolute concentration. Note that *D*_PS_ has the units of a diffusion coefficient (e.g., m^2^·s^−1^).

The term “diffusiophoresis” was originally introduced by Derjaguin in 1947 to describe the motion of large colloidal particles in the presence of concentration gradients of small ions and molecules [15]. It was then investigated by Andersen and Prieve [15,16,26], especially in connection with deposition rates of latex particles onto porous membranes. They also derived mathematical expressions for diffusiophoretic mobilities by employing hydrodynamic models [20,27,28]. These are the same types of models that were previously employed for describing electrophoretic mobilities (Henry’s equation) [17] and also the friction encountered by Brownian particles (Stokes’ law) [29]. With the advent of microfluidic technologies, many more diffusiophoresis studies were reported, showing the value of this transport phenomenon in manipulating the motion of colloidal particles. In the chemistry field, the term “chemotaxis” sometimes replaces “diffusiophoresis”, emphasizing similarities between the migration of colloidal particles and the movement of living organisms in response to gradients of nutrient or toxic molecules [5,30,31].

Experimental studies have demonstrated that diffusiophoresis promotes particle mixing, focusing and separation in microfluidics [18,19,32,33,34], particle entrainment in dead-end pores [8,18], particle deposition on porous membranes [20,35]. Moreover, diffusiophoresis was employed to achieve membraneless water filtration [22] and remove staining agents from fibrous materials [36]. The reader should, for example, consult two recent review articles by Shim [13] and Velegol [12] describing the various applications of diffusiophoresis in mass-transfer problems.

An expert in the field of transport phenomena may realize that diffusiophoresis should be associated with the phenomenon of cross-diffusion encountered in multicomponent mixtures [37,38,39,40,41,42,43,44,45,46]. Cross-diffusion is described by employing the framework of non-equilibrium thermodynamics [47,48], which was derived primarily from the statistical mechanical investigations of Onsager in 1931 [49,50]. It has been investigated by several scientists in the fields of physical chemistry [37,39,51,52,53,54,55] and chemical engineering [45,46,56,57]. Cross-diffusion coefficients are typically defined by writing Fickian relations [58] in which the flux of a solute not only depends on its concentration gradient (main-term diffusion coefficients) but also on the concentration gradient of other solutes (cross-term diffusion coefficients) [41]. Thus, multicomponent diffusion in a ternary system is described by a 2 × 2 diffusion-coefficient matrix. The two main terms describe the diffusion of the two solutes due to their own concentration gradients, while the two cross-terms describe the diffusion of a solute due to the concentration gradient of the other solute. Thus, a diffusiophoretic mobility should be closely related to the cross-term diffusion coefficient describing flux of a large solute due to the concentration gradient of the relatively small solute.

Multicomponent-diffusion coefficients can be experimentally determined. The well-established Taylor dispersion method [38,43,59,60] and interferometric techniques [37,40,61,62] have been successfully employed for the measurements of these transport parameters. Thus, the connection of diffusiophoresis to multicomponent diffusion is important because it provides the basis for determining diffusiophoretic mobilities from measurements of multicomponent-diffusion coefficients. This was first highlighted by Shaeiwitz and Lechnick in 1983 [63]. It is surprising that previous studies on diffusiophoresis were disconnected from work on multicomponent diffusion. This is even more striking considering that the theoretical electrokinetic models describing diffusiophoresis [13,15] of charged particles are fundamentally the same as those at the heart of Nernst–Planck and related Nernst–Hartley equations [52,64,65,66], which are employed for modeling multicomponent diffusion in dilute electrolyte mixtures. Presumably, diffusiophoresis has been regarded as a transport phenomenon separated from cross-diffusion because it pertains to large colloidal particles and, consequently, was originally introduced within the framework of hydrodynamics. On the other hand, the phenomenon of multicomponent diffusion, which covers the case of solutes of various sizes, is described by employing non-equilibrium thermodynamics. It is worth mentioning that a similar separation existed in the past between multicomponent diffusion coefficients and the collective diffusion coefficient determined by dynamic light scattering (DLS) [67,68]. The extension of multicomponent-diffusion studies to mixtures containing macromolecules [69] or micelles [70] then prompted more attention to the connection between DLS and multicomponent diffusion. It is now well known that the DLS diffusion coefficient represents the lower eigenvalue of the 2×2 diffusion-coefficient matrix of ternary liquid mixtures of colloidal particles and a cosolute of low molar weight [69,70,71].

We know that diffusiophoresis should be regarded as a special case of cross-diffusion [71,72]. It describes the cross-diffusion of a solute that 1) is large compared to the other components of the mixture and 2) is present at low concentration in the mixture. There is some gray area when it comes to considering the size and concentration scales relevant to diffusiophoresis. In relation to size, the same type of uncertainty is encountered in the application of the Stokes–Einstein equation with no-slip boundary condition [73]. Since this is typically applied to particles with a radius of ≈1 nm and larger, it is reasonable to assume that this size range also pertains to diffusiophoresis. In other words, diffusiophoresis is a phenomenon that is relevant to proteins, polymers, micelles and other nanoparticles of comparable (1–10 nm) or lager (≈100 nm) size. In relation to concentration, one could readily enunciate that diffusiophoresis represents cross-diffusion in the limit of zero solute concentration. However, while this definition is conceptually correct, it is possible that the infinite-dilute limit is difficult to attain experimentally due to the complex nature of colloidal particles [23,74]. For example, there is a range of supramolecular aggregates, such as micelles, that disassemble into free units as solute concentration approaches zero [75]. From a practical point of view, it is convenient to identify sufficiently low concentrations at which cross-term diffusion coefficients can be successfully measured and verify that the extracted diffusiophoretic mobility is independent of particle concentration within the experimental error. Fortunately, as it will be discussed later, non-equilibrium thermodynamics shows that the cross-term diffusion coefficient associated with diffusiophoresis does not explicitly depend on the thermodynamic non-ideality of colloidal particles. In contrast, thermodynamic non-ideality is known to cause a significant concentration dependence of the DLS diffusion coefficient [23,68,76,77]. Note that diffusiophoresis of a colloidal particle is explicitly related to how particle chemical potential depends on the concentration of the cosolute [25]. Indeed, a gradient of cosolute concentration causes a chemical-potential gradient of the colloidal particle, which may be then responsible for its migration from high to low chemical potential.

The thermodynamic effect of cosolutes on colloidal particles has been extensively investigated in the case of proteins by introducing thermodynamic parameters known as *preferential-interaction coefficients* [78,79,80,81,82,83,84]. These quantify the thermodynamic affinity of a protein for either the solvent (water) or the cosolute (salt or osmolyte). Related experimental and theoretical studies have played a crucial role in understanding equilibrium dialysis [85], protein solubility and crystallization [86,87,88], conformational stability [89], Hofmeister series [90,91] and modulation of biochemical reactions [79]. The connection of preferential-interaction coefficients to diffusiophoresis will also be discussed in this paper.

As previously mentioned, two cross-term diffusion coefficients are needed to describe a ternary system, with one of them describing diffusiophoresis. The other cross-term, which describes cosolute diffusion due to the concentration gradient of colloidal particles, will be denoted as cosolute “osmotic diffusion” [25,72,92]. This term was first introduced by Toor in 1957 as an alternative name for cross-diffusion in multicomponent mixtures [93]. Using different names (“diffusiophoresis” and “osmotic diffusion”) facilitates distinction between the two related cross-diffusion processes. Interestingly, as will be discussed below, cosolute osmotic diffusion is more closely related to preferential-interaction coefficients than particle diffusiophoresis [25,72,92]. Thus, it plays an important role in understanding the thermodynamic component of particle diffusiophoresis and identifying the underlying mechanisms responsible for this transport process [25,92].

The following sections of this paper intend to provide a formulation of diffusiophoresis by employing the formalism of multicomponent diffusion and non-equilibrium thermodynamics. Representative experimental diffusiophoresis data previously obtained on two different macromolecules, polyethylene glycol (PEG) [25,92,94] and lysozyme [66,71,72,95], will also be reviewed. These sections will be organized in the following way. In Section 2, the theoretical background of non-equilibrium thermodynamics that is relevant to multicomponent diffusion in ternary systems is reviewed. In Section 3 and Section 4, the formulation of non-equilibrium thermodynamics is used to describe both diffusiophoresis and osmotic diffusion. Specifically, reduced diffusiophoresis and osmotic diffusion coefficients will be introduced. Their connection to preferential-interaction coefficients will also be discussed. In Section 5, the connection of preferential-interaction coefficients to the mechanisms of preferential solvation of colloidal particles and Donnan equilibrium [71,73] for charged particles will be discussed. In Section 6, the Nernst–Planck equations for ternary electrolyte mixtures [52,64,71] are revisited to derive mathematical expressions for diffusiophoresis and osmotic diffusion coefficients. In Section 7, the determination of these two transport parameters from measurements of multicomponent-diffusion coefficients by employing the Gosting diffusiometer operating in the Rayleigh interferometry method will be discussed. In Section 8, representative experimental results on diffusiophoresis of PEG [25,92,94], a neutral hydrophilic macromolecule in the presence of salts and osmolytes will be presented. In Section 9, a useful model [25] based on preferential hydration will be discussed. This has been employed to describe the experimental results of PEG. In Section 10, the experimental results on diffusiophoresis of lysozyme [66,71,72,95], a positively charged protein, in the presence of salts will be presented. In Section 11 and Section 12, the experimental results on lysozyme will be discussed by considering both the electrophoretic (Section 11) and preferential-hydration (Section 12) mechanisms responsible for salt-induced protein diffusiophoresis.

## 2. Diffusion, Onsager Transport Coefficients and Reference Frames

In this section, the words “colloidal particle” or simply “particle” will be used to generally indicate macromolecules (proteins, polymers), supramolecular aggregates (micelles, vesicles) and nanoparticles (silica, polystyrene latex), with characteristic sizes ranging from 1 nm to 1 mm. The symbols “P” for colloidal particle, “S” for cosolute (e.g., salt or osmolyte) and “W” for solvent (e.g., water) will be employed.

In the absence of advection and chemical reactions, isothermal and isobaric diffusion in a ternary particle–cosolute–solvent liquid mixture is described by two linear laws relating the fluxes of the two solutes (P and S) to the two independent thermodynamic driving forces, the gradients of particle and cosolute chemical potentials. We have the following [51,96]:

(2a) $$-J_{P}=L_{\mathrm{PP}}\;\nabla \;\mu_{P}+L_{\mathrm{PS}}\;\nabla \;\mu_{S}$$

(2b) $$-J_{S}=L_{\mathrm{SP}}\;\nabla \;\mu_{P}+L_{\mathrm{SS}}\;\nabla \;\mu_{S}$$

where *J*_P_ and *J*_S_ are the molar fluxes of particle and cosolute, and *μ*_P_ and *μ*_S_ the corresponding chemical potentials. The matrix of the four *L_ij_*s (with *i*,*j* = P,S) is the Onsager transport coefficient [51,53]. Main-term coefficients, *L*_PP_ and *L*_SS_, describe the flux of solutes due to their own concentration gradients, while cross-term coefficients, *L*_PS_ and *L*_SP_, describe the flux of a solute due to the concentration gradient of the other solute.

**Figure 2:**
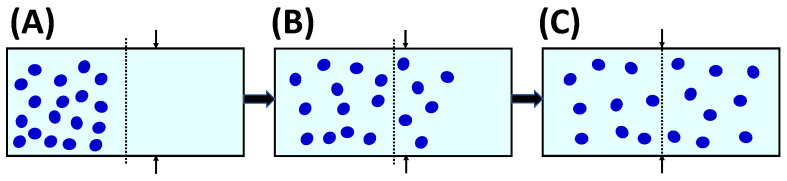
Diffusion of a solute inside a cell (rectangular boxes) occurring from high to low solute concentration. Solute particles are explicitly shown (blue particles), while solvent molecules are described as a uniform background for simplicity. The three boxes (**A**–**C**) indicate three representative times during the diffusion process. Box (**A**) represents the initial state of the solute–solvent mixture, while box (**C**) indicates its final equilibrium state (homogeneous mixture). The vertical dashed line indicates the position of the center of volume. This remains unchanged if the mixing volume is zero (i.e., if isobaric mixing is also isochoric). In the solvent-fixed reference frame, diffusion of cosolute is evaluated with respect to the solvent center of mass, which is indicated by the evolving position of the arrows. The solvent center of mass shifts from right to left during diffusion. The difference between solvent- and volume-fixed reference frames increases with solute volume fraction and vanishes in the limit of zero solute concentration. In this limit, both reference frames yield the same diffusion coefficient, which is also known as the tracer-diffusion coefficient of solute particles.

In Equation (2a,b), molar fluxes can also be rewritten as *J*_P_ = *C*_P_·*v*_P_ and *J*_S_ = *C*_S_·*v*_S_ [39,96], where *C*_P_ and *C*_S_ are the molar concentrations of particle and cosolute, while *v*_P_ and vs. are the corresponding diffusion velocities, which must be defined with respect to a reference frame. In the *volume*-fixed reference frame, *v*_P_ and vs. are defined relative to the center of volume of the ternary mixture, which corresponds to the external laboratory coordinate if mixing can be assumed to occur in isochoric conditions (i.e., the volume of mixing is negligible). In the *solvent*-fixed reference frame, *v*_P_ and vs. are defined relative to solvent diffusion, i.e., solvent diffusion velocity is set to be zero [39,96]. Both reference frames are qualitatively described in Figure 2 in the case of a binary mixture for simplicity.

According to non-equilibrium thermodynamics, the matrix of the four *L_ij_*s becomes symmetric in the solvent-fixed reference frame, with

(3) $$L_{\mathrm{PS}}=L_{\mathrm{SP}}$$

representing the Onsager reciprocal relation (ORR) [51,53]. Thus, Equation (2a,b) are usually employed to describe diffusion in the solvent-fixed reference frame. Note that diffusive transport in ternary mixtures is characterized by three independent transport coefficients.

For completeness, it is important to note that diffusion can be equivalently described by inverting Equation (2a,b). In this description, the particle thermodynamic driving force, ▽*μ*_P_, is written as a linear combination of the velocity differences, *v*_P_-*v*_W_ and *v*_P_-*v*_S_, while cosolute thermodynamic driving force, ▽*μ*_S_, is a linear combination of the velocity differences, *v*_S_-*v*_W_ and *v*_S_-*v*_P_ [97]. In the field of chemical engineering, this description corresponds to the well-known Stefan–Maxwell equations [45,46,57,72,98]. The convenience of this formalism is that the coefficients of these linear combinations can be interpreted as frictional coefficients, which are also independent of the reference frame [97,99]. There are three independent friction coefficients characterizing particle–solvent, cosolute–solvent and particle–cosolute frictional interaction. These could be, in principle, experimentally determined from measurements on the three corresponding binary mixtures and assumed to be the same in the ternary mixture. However, since the particle–cosolute binary system is not accessible experimentally, Stefan–Maxwell equations are not particularly valuable for diffusiophoresis. Moreover, multicomponent-diffusion experiments on aqueous PEG in the presence of several cosolutes (osmolytes and salts) show that the PEG–cosolute frictional coefficient in water is negative [25,92,99]. This implies that its physical interpretation as an actual friction between PEG and cosolute components is misleading. Thus, Stefan–Maxwell equations do not seem to provide noteworthy benefits over Equation (2a,b) and will not be further considered below.

According to Equation (2a,b), molar fluxes, *J*_P_ and *J*_S_, are related to the gradient of chemical potentials, ▽*μ*_P_ and ▽*μ*_S_. Although these are the actual thermodynamic driving forces of diffusion, it is practically convenient to describe diffusion as caused by concentration gradients, ▽*C*_P_ and ▽*C*_S_. This description is just a generalization of the well-known Fick’s law [58,100], normally encountered when describing binary mixtures [51]. The 2 × 2 matrix of Fickian diffusion coefficients is introduced by the following:

(4a) $$-J_{P}=D_{\mathrm{PP}}\;\nabla \;C_{P}+D_{\mathrm{PS}}\;\nabla \;C_{S}$$

(4b) $$-J_{S}=D_{\mathrm{SP}}\;\nabla \;C_{P}+D_{\mathrm{SS}}\;\nabla \;C_{S}$$

where the four *D_ij_*s (with *I*,*j* = P,S) are denoted as multicomponent-diffusion coefficients (or ternary diffusion coefficients in this case). Main-term diffusion coefficients, *D*_PP_ and *D*_SS_, describe the flux of solutes due to their own concentration gradients, while cross-term diffusion coefficients, *D*_PS_ and *D*_SP_, describe the flux of a solute due to the concentration gradient of the other solute. Note that *D*_PS_ in Equation (4a) is directly proportional to *C*_P_ with *D*_PS_ = 0 at *C*_P_ = 0 [72]. Thus, it is more appropriate to consider the ratio *D*_PS_/*C*_P_ when examining particle diffusiophoresis. Indeed, the value of this ratio becomes virtually independent of *C*_P_ in dilute colloidal solutions [72].

We can write Equation (4a,b) with respect to any reference frame, but it is convenient to define them with respect to solvent-fixed reference frame so that we can more directly connect Equation (4a,b) with a symmetric matrix of Onsager transport coefficients. Comparison of Equation (2a,b) with Equation (4a,b) yields the following [53]:

(5a) $$D_{\mathrm{PP}}=L_{\mathrm{PP}}\mu_{\mathrm{PP}}+L_{\mathrm{PS}}\mu_{\mathrm{SP}}$$

(5b) $$D_{\mathrm{PS}}=L_{\mathrm{PP}}\mu_{\mathrm{PS}}+L_{\mathrm{PS}}\mu_{\mathrm{SS}}$$

(5c) $$D_{\mathrm{SP}}=L_{\mathrm{SP}}\mu_{\mathrm{PP}}+L_{\mathrm{SS}}\mu_{\mathrm{SP}}$$

(5d) $$D_{\mathrm{SS}}=L_{\mathrm{SP}}\mu_{\mathrm{PS}}+L_{\mathrm{SS}}\mu_{\mathrm{SS}}$$

where *μ_ij_* ≡ (𝜕*μ_i_*/𝜕*C_j_*)*_Ck_*_,*k*≠*j*_ and the partial-derivative subscripts denoting temperature and pressure are omitted to alleviate notation. The four *μ_ij_*s in Equation (5a–d) are thermodynamically linked by the following [53,101]:

(6) $$\mu_{\mathrm{SP}}(1-C_{P}{\bar{V}}_{P})+\mu_{\mathrm{PP}}C_{P}{\bar{V}}_{S}=\mu_{\mathrm{PS}}(1-C_{S}{\bar{V}}_{S})+\mu_{\mathrm{SS}}C_{S}{\bar{V}}_{P}$$

where ${\bar{V}}_{P}$ and ${\bar{V}}_{S}$ are particle and cosolute partial molar volumes, respectively.

The limit of *C*_P_→0 is particularly important for particle diffusiophoresis. In this limit, *D*_PP_ becomes the particle tracer-diffusion coefficient, *D*_P_(*C*_S_), while *D*_SS_ is the cosolute diffusion coefficient of the binary cosolute–solvent system in the solvent-fixed reference frame, *D*_S_(*C*_S_). We have the following:

(7a) $$D_{P}\equiv \underset{C_{P}\to 0}{\lim}D_{\mathrm{PP}}=\underset{C_{P}\to 0}{\lim}(L_{\mathrm{PP}}\mu_{\mathrm{PP}})=\underset{C_{P}\to 0}{\lim}\frac{RTL_{\mathrm{PP}}}{C_{P}}$$

(7b) $$D_{S}\equiv \underset{C_{P}\to 0}{\lim}D_{\mathrm{SS}}=\underset{C_{P}\to 0}{\lim}(L_{\mathrm{SS}}\mu_{\mathrm{SS}})$$

where *T* is the absolute temperature, R is the ideal gas constant, and we have also used *μ*_PP_→ R*T*/*C*_P_. Note that *L*_PP_→0 and *L*_PS_→0 while *L*_PP_/*C*_P_ and *L*_PS_/*L*_PP_ remain finite in the limit of *C*_P_→0.

Multicomponent-diffusion coefficients are typically measured in the volume-fixed frame [39,53,96]. The solvent-fixed diffusion coefficients, $D_{ij}$, can then be calculated from the corresponding volume-fixed diffusion coefficients, $D_{ij}^{V}$, by the following [39,51,102]:

(8a) $$\frac{D_{\mathrm{PP}}-D_{\mathrm{PP}}^{V}}{C_{P}}=\frac{D_{\mathrm{SP}}-D_{\mathrm{SP}}^{V}}{C_{S}}=\frac{{\bar{V}}_{P}D_{\mathrm{PP}}^{V}+{\bar{V}}_{S}D_{\mathrm{SP}}^{V}}{1-C_{P}{\bar{V}}_{P}-C_{S}{\bar{V}}_{S}}$$

(8b) $$\frac{D_{\mathrm{SS}}-D_{\mathrm{SS}}^{V}}{C_{S}}=\frac{D_{\mathrm{PS}}-D_{\mathrm{PS}}^{V}}{C_{P}}=\frac{{\bar{V}}_{P}D_{\mathrm{PS}}^{V}+{\bar{V}}_{S}D_{\mathrm{SS}}^{V}}{1-C_{P}{\bar{V}}_{P}-C_{S}{\bar{V}}_{S}}$$

In the limit of *C*_P_→0, Equation (8a,b) yield the following:

(9a) $$\underset{C_{P}\to 0}{\lim}D_{\mathrm{PP}}^{V}=\underset{C_{P}\to 0}{\lim}D_{\mathrm{PP}}=D_{P}$$

(9b) $$\underset{C_{P}\to 0}{\lim}D_{\mathrm{SS}}^{V}=D_{S}(1-C_{S}{\bar{V}}_{S})$$

(9c) $$\underset{C_{P}\to 0}{\lim}\frac{D_{\mathrm{PS}}}{C_{P}}-\underset{C_{P}\to 0}{\lim}\frac{D_{\mathrm{PS}}^{V}}{C_{P}}={\bar{V}}_{S}D_{S}$$

(9d) $$\underset{C_{P}\to 0}{\lim}\frac{D_{\mathrm{SP}}}{D_{\mathrm{SS}}}-\underset{C_{P}\to 0}{\lim}\frac{D_{\mathrm{SP}}^{V}}{D_{\mathrm{SS}}^{V}}=\frac{{\bar{V}}_{P}D_{P}}{(1-C_{S}{\bar{V}}_{S})D_{S}}$$

The conversion terms between the two reference frames are typically small and may be comparable with experimental error in the case of cross-term diffusion coefficients.

## 3. Particle Diffusiophoresis from Non-Equilibrium Thermodynamics

In this section, particle diffusiophoresis is described using the framework of non-equilibrium thermodynamics in the solvent-fixed reference frame. Since the actual thermodynamic driving force of this transport process is the gradient of cosolute chemical potential, we need to replace ▽ln*C*_S_ in Equation (1) with ▽*μ*_S_. In the limit of *C*_P_→0, we can write the following:

(10) $$\frac{\;\nabla \;\mu_{S}}{RT}=\nu_{S}y_{S}\;\nabla \;\ln C_{S}$$

where *ν*_S_ is the number of cosolute particles (e.g., *ν*_S_ = 1 for non-electrolytes, *ν*_S_ = 2 for symmetric electrolytes such as NaCl or MgSO_4_ and *ν*_S_ = 3 for asymmetric electrolytes such as MgCl_2_ or Na_2_SO_4_) and *y*_S_ is a thermodynamic factor (known for many cosolutes [103,104,105,106,107]) characterizing thermodynamic non-ideality of the binary cosolute–water system, with *y*_S_ = 1 in the limit of *C*_S_→0. Since *D*_PS_ has the units of a diffusion coefficient, it is also convenient to consider the unitless ratio, *D*_PS_/*D*_P_, which describes the magnitude of diffusiophoresis compared to particle Brownian mobility. Note that this ratio is also independent of fluid viscosity because both *D*_PS_ and *D*_P_ are inversely proportional [15,27,30,73,108] to the fluid viscosity coefficient. We, therefore, describe diffusiophoresis by writing the following:

(11) $$v_{P}=-D_{P}\left(\;\nabla \;\ln C_{P}+{\hat{D}}_{\mathrm{PS}}\;\frac{\;\nabla \;\mu_{S}}{RT}\right)$$

where the second term in parenthesis characterizes particle diffusiophoresis with ${\hat{D}}_{\mathrm{PS}}$ being the reduced diffusiophoresis coefficient [25,39,102]. The first term in Equation (11) represents the restoring Brownian entropic force. The reduced coefficient, ${\hat{D}}_{\mathrm{PS}}$, is related to *D*_PS_ by the following:

(12) $$D_{\mathrm{PS}}=D_{P}\nu_{S}y_{S}\;{\hat{D}}_{\mathrm{PS}}$$

where *ν*_S_*y*_S_ represents the conversion factor from ∇ln*C*_S_ to ∇*μ*_S_/R*T* (see Equation (10)). Thus, the convenience of ${\hat{D}}_{\mathrm{PS}}$ compared to *D*_PS_ is that it does not explicitly depend on the stoichiometry and thermodynamic non-ideality of cosolute. Henceforth, diffusiophoresis will be described using ${\hat{D}}_{\mathrm{PS}}$, recognizing that it can be readily converted into *D*_PS_ by employing Equation (12).

**Figure 3:**
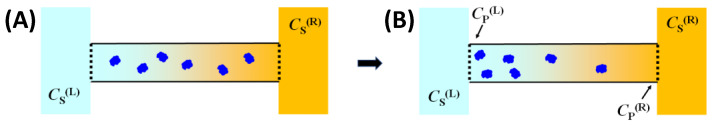
Schematic diagrams showing a horizontal tube connected to two cosolute–solvent reservoirs with constant cosolute concentrations, *C*_S_^(L)^ and *C*_S_^(R)^, through two semipermeable membranes. (**A**) The tube is initially at uniform particle concentration. (**B**) After reaching the steady-state condition (right diagram), diffusiophoresis causes a difference in particle concentration at the tube extremities (*C*_P_^(L)^ and *C*_P_^(R)^). Colloidal particles are explicitly shown, while cosolute and solvent molecules are described as a uniform background for simplicity, with the color gradient representing the cosolute gradient.

There is a simple case in which values of ${\hat{D}}_{\mathrm{PS}}$ have a direct physical interpretation. Specifically, we may consider a steady-state diffusion process that is appropriate for colloidal particles in the presence of relatively small ions and molecules of cosolute and solvent components [92]. We shall neglect cosolute thermodynamic non-ideality (*y*_S_ = 1) and small corrections due to changes in the reference frame for simplicity. As illustrated in Figure 3, a horizontal tube positioned between *x* = 0 and *x* = *l* is sandwiched between two reservoirs consisting of two different binary cosolute−solvent solutions, with cosolute concentrations, *C*_S_^(L)^ (left compartment, *x* ≤ 0) and *C*_S_^(R)^ > *C*_S_^(L)^ (right compartment, *x* ≥ *l*), respectively. Two membranes, not permeable to colloidal particles, seal the two tube extremities. This type of geometries, which is needed to establish steady-state concentration gradients of cosolutes, has been indeed implemented in “H-type” microfluidic devices to investigate bacterial chemotaxis [109,110] and particle diffusiophoresis [111].

In steady-state conditions, we must have *v*_P_ = 0 throughout the tube because of the semipermeable membranes. Hence, Equation (11) becomes the following:

(13) $$\frac{d\ln C_{P}}{d(\nu_{S}C_{S})}=-\;\frac{{\hat{D}}_{\mathrm{PS}}}{C_{S}}$$

where *ν*_S_*C*_S_ is cosolute osmolarity. This steady-state process is analogous to equilibrium sedimentation in which sedimentation due to external gravitational field is counter-balanced by the restoring entropic force associated with Brownian diffusion.

As we shall see later, ${\hat{D}}_{\mathrm{PS}}$/*C*_S_ is essentially a constant for neutral particles [92]. In this case, integration of Equation (13) shows that ln[*C*_P_^(L)^/*C*_P_^(R)^] ≈ (${\hat{D}}_{\mathrm{PS}}$/*C*_–_)·*ν*_S_*C*_P_^(L)^ − *C*_P_^(R)^]. For example, when we set the difference in cosolute osmolari–y t*ν*_S_[*C*_P_^(L)^ − *C*_P_^(R)^] = 1 M, we obtain *C*_P_^(L)^/*C*_P_^(R)^ ≈ 2.7 if ${\hat{D}}_{\mathrm{PS}}$/*C*_S_ ≈ 1 M^−1^ and *C*_P_^(L)^/*C*_P_^(R)^ ≈ 150 if ${\hat{D}}_{\mathrm{PS}}$/*C*_S_ ≈ 5 M^−1^. For charged particles, at salt concentrations of the order of 1 mM, we can approximately assume that ${\hat{D}}_{\mathrm{PS}}$ is a constant. In this second case, integration shows that ln[*C*_P_^(L)^/*C*_P_^(R)^] ≈ ${\hat{D}}_{\mathrm{PS}}\cdot$ ln[*C*_S_^(L)^/*C*_S_^(R)^]*^ν^*^S^. When we then set the ratio in salt concentration to *C*_S_^(L)^/*C*_S_^(R)^ = 100 (e.g., *C*_S_^(L)^ = 0.1 mM and *C*_S_^(R)^ = 10 mM) with *ν*_S_ = 2, we obtain *C*_P_^(L)^/*C*_P_^(R)^≈100 if ${\hat{D}}_{\mathrm{PS}}$ ≈ 0.5.

We now turn our attention to the link between the diffusiophoresis coefficient, ${\hat{D}}_{\mathrm{PS}}$, and Onsager transport coefficients. In the limit of *C*_P_→0, Equation (7a) and *J*_P_ = *C*_P_·*v*_P_ can be used to rewrite Equation (4a) in the following way [23]:

(14) $$v_{P}=-D_{P}\left(\frac{\nabla \mu_{P}}{RT}-\;\lambda \frac{\nabla \mu_{S}}{RT}\right)$$

where *λ* is a reduced hydrodynamic coefficient defined by the following:

(15) $$\lambda \equiv -\underset{C_{P}\to 0}{\lim}\frac{L_{\mathrm{PS}}}{L_{\mathrm{PP}}}$$

In Equation (14), *λ* describes particle migration in response to the gradient of cosolute chemical potential when ▽*μ*_P_ = 0. As it will be shown below, this does not correspond to ▽ln*C*_P_ = 0. Note that the negative sign in Equation (15) is just a convention, justified by *λ* being a positive hydrodynamic coefficient for colloidal particles that preferentially interact with solvent [92]. To link ${\hat{D}}_{\mathrm{PS}}$ to *λ*, we need to relate ▽*μ*_P_ to ▽ln*C*_P_. This is achieved by considering the differential of *μ*_P_(*C*_P_, *μ*_S_):

(16) $$\frac{\nabla \mu_{P}}{RT}=\nabla \ln C_{P}+\gamma \;\frac{\nabla \mu_{S}}{RT}$$

where

(17) $$\gamma \equiv \underset{C_{P}\to 0}{\lim}{\left(\frac{𝜕\mu_{P}}{𝜕\mu_{S}}\right)}_{C_{P}}=\underset{C_{P}\to 0}{\lim}\frac{\mu_{\mathrm{PS}}}{\mu_{\mathrm{SS}}}$$

is a preferential-interaction coefficient. If the expression of ▽*μ*_P_ given by Equation (16) is inserted into Equation (14), we can finally deduce the following [71,95]:

(18) $${\hat{D}}_{\mathrm{PS}}=\;\gamma -\lambda$$

In summary, non-equilibrium thermodynamics is used to write particle diffusiophoresis coefficient, ${\hat{D}}_{\mathrm{PS}}$, as the difference between a thermodynamic component (*γ*) and a transport component (*λ*). It has been experimentally shown that the magnitude of *λ* is typically comparable with that of *γ* [92]. This implies that both thermodynamic and hydrodynamic interactions are equally important in shaping the behavior of ${\hat{D}}_{\mathrm{PS}}$.

## 4. Cosolute Osmotic Diffusion from Non-Equilibrium Thermodynamics

**Figure 4:**
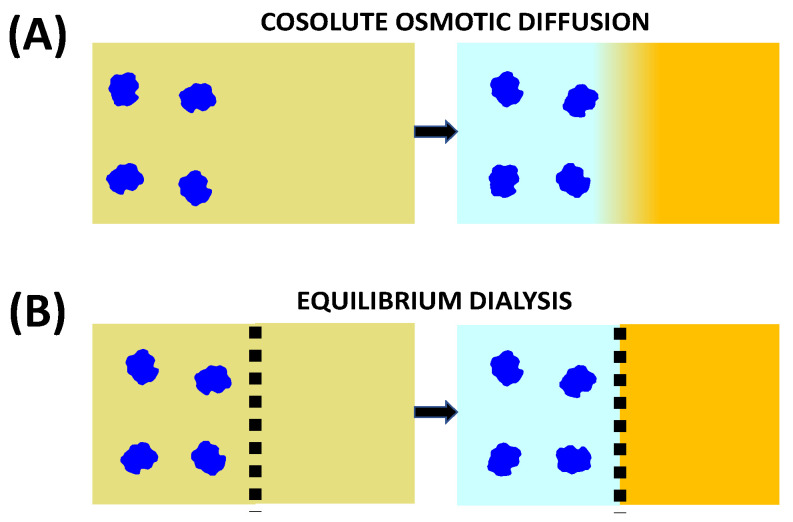
(**A**) Cosolute osmotic diffusion induced from high to low concentration of colloidal particles. (**B**) Equilibrium dialysis setup (bottom diagrams). Here, two compartments containing a ternary particle–cosolute–solvent system (left side) and a binary cosolute–solvent system (right side) are separated by a membrane (dashed line) not permeable to particles. Due to cosolute osmotic diffusion, a difference in cosolute concentration is established between the two compartments. Colloidal particles are explicitly shown, while cosolute and solvent molecules are described as a uniform background for simplicity, with the color gradient representing the cosolute gradient. Note that solvent chemical potential, not pressure, is constant across the membrane in equilibrium dialysis, while cosolute osmotic diffusion is defined for constant pressure. There is a small difference between the two cases, which is ignored in our qualitative comparison for simplicity.

Cosolute osmotic diffusion describes cosolute diffusion induced by a concentration gradient of colloidal particles. As in the case of diffusiophoresis, a reduced coefficient is introduced for osmotic diffusion. Since the diffusion of colloidal particles is slow compared to that of cosolute, it is also convenient to consider the hypothetical limit in which particle mobility is negligible compared to cosolute mobility [71]. In this case, a particle concentration gradient will dissipate after a time that is infinitely long compared to that needed for cosolute diffusion to reach a quasi-equilibrium condition. This condition is achieved when the osmotic diffusion rate, *D*_SP_∇*C*_P_, is counter-balanced by the cosolute diffusion rate, *D*_SS_∇*C*_S,_ in Equation (4b). Thus, the ratio *D*_SP_/*D*_SS_ essentially describes the equilibrium distribution of cosolute along a quasi-static concentration gradient of particles. This ratio is analogous to an equilibrium constant of a reversible reaction, which is the ratio of forward and backward kinetic constants. Cosolute osmotic diffusion is illustrated in Figure 4. Here, we can also appreciate that this transport process is also related to cosolute partitioning occurring in equilibrium dialysis. Specifically, we can consider two compartments containing a ternary particle–cosolute–solvent system and a binary cosolute–solvent system in chemical equilibrium with respect to the cosolute component through a membrane that is not permeable to particles. The difference in particle concentration between the two compartments is responsible for cosolute osmotic diffusion across the membrane, thereby leading to a difference in cosolute concentration between the two compartments at equilibrium. The connection of equilibrium dialysis to cosolute osmotic diffusion emerges from the fact that cosolute partitioning between the two compartments can also be achieved in the absence of a membrane, provided that the Brownian mobility of the colloidal particles is negligible compared to cosolute mobility.

Since the ratio *D*_SP_/*D*_SS_ is closely connected to cosolute partitioning at equilibrium, it is convenient to define the unitless osmotic diffusion coefficient [25,71]:

(19) $${\hat{D}}_{\mathrm{SP}}\equiv \underset{C_{P}\to 0}{\lim}\frac{D_{\mathrm{SP}}}{D_{\mathrm{SS}}}$$

We can use non-equilibrium thermodynamics to rigorously relate this coefficient to cosolute partitioning. Specifically, we first take the ratio of the expressions of *D*_SP_ (Equation (5c)) and *D*_SS_ (Equation (5d)). We then consider that we have: *D*_SS_→*L*_SS_*μ*_SS_ in the limit of *C*_P_→0 (see Equation (7b)). This allows us to write *D*_SP_/*D*_SS_→(*L*_SP_*μ*_PP_)/(*L*_SS_*μ*_SS_)+ *μ*_SP_/*μ*_SS_. As will be further discussed below, it is the thermodynamic ratio, *μ*_SP_/*μ*_SS_, that describes cosolute partitioning. The product, *L*_SP_*μ*_PP_, can be rewritten as (*L*_PP_*μ*_PP_)(*L*_PS_/*L*_PP_), where we have also used the ORR (Equation (3)). According to Equation (7a,b), we can then write (*L*_PP_*μ*_PP_)/(*L*_SS_*μ*_SS_)→*D*_P_/*D*_S_. We finally obtain the following:

(20) $${\hat{D}}_{\mathrm{SP}}=C_{\mathrm{SP}}-\alpha \lambda$$

where

(21) $$C_{\mathrm{SP}}\equiv -\;\;\underset{C_{P}\to 0}{\lim}{\left(\frac{𝜕C_{S}}{𝜕C_{P}}\right)}_{\mu_{S}}=\underset{C_{P}\to 0}{\lim}\frac{\mu_{\mathrm{SP}}}{\mu_{\mathrm{SS}}}$$

and we have also used (𝜕*μ*_S_/𝜕*C*_P_)*_C_*_S_ = −(𝜕*μ*_S_/𝜕*C*_S_)*_C_*_P_(𝜕*C*_S_/𝜕*C*_P_)*_μ_*_S_. The thermodynamic factor, *C*_SP_, characterizes the effect of particle concentration on cosolute concentration at equilibrium. The negative sign in the definition of *C*_SP_ is just a convention. It ensures that this parameter assumes positive values for colloidal particles that preferentially interact with solvent. Integration of *C*_SP_ with respect to *C*_P_ characterizes cosolute partitioning. In Equation (20), we have also defined the following:

(22) $$\alpha \equiv \frac{D_{P}}{D_{S}}$$

as the particle-to-salt diffusion ratio, with typically *α* ≈ 0.1 or less. In Equation (22), *αλ* is small compared to *C*_SP,_ and ${\hat{D}}_{\mathrm{SP}}$ ≈ *C*_SP_ is a suitable approximation, which is consistent with our previous discussion. Indeed, we can state that ${\hat{D}}_{\mathrm{SP}}$→*C*_SP_ in the limit of *α*→0. Note that Equation (3) allows us to introduce *λ* not only in Equation (18) for ${\hat{D}}_{\mathrm{PS}}$ but also in Equation (20) for ${\hat{D}}_{\mathrm{SP}}$. In other words, the ORR connects ${\hat{D}}_{\mathrm{SP}}$ with ${\hat{D}}_{\mathrm{PS}}$. However, the validity ORR is not critical because *αλ* in Equation (23) is small. To fully link ${\hat{D}}_{\mathrm{SP}}$ and ${\hat{D}}_{\mathrm{SP}}$, we also need to relate *C*_SP_ in Equation (20) to *g* in Equation (18). This is achieved by determining the expression of *μ*_SP_/*μ*_SS_ from Equation (6) in the limit of *C*_P_→0. We obtain the following [53,71,80,101,102]:

(23) $$C_{\mathrm{SP}}\;=\;(1-C_{S}{\bar{V}}_{S})\;\gamma \;+C_{S}{\tilde{V}}_{P}$$

where ${\tilde{V}}_{P}$ ≡ ${\bar{V}}_{P}$ − (*ν*_S_*y*_S_)^−1^${\bar{V}}_{S}$. Note that ${\tilde{V}}_{P}$ = ${\bar{V}}_{P}$ is an excellent approximation because ${\bar{V}}_{S}$ is significantly smaller than ${\bar{V}}_{P}$. It is Equation (23) that critically connects ${\hat{D}}_{\mathrm{PS}}$ with ${\hat{D}}_{\mathrm{SP}}$. Since both coefficients are related to *γ* and *λ*, measurements of ${\hat{D}}_{\mathrm{PS}}$ and ${\hat{D}}_{\mathrm{SP}}$ can be used to extract these two fundamental parameters. Although *γ* and *λ* can be rigorously calculated by combining Equations (18), (20) and (23), it is important to appreciate that ${\hat{D}}_{\mathrm{SP}}$ ≈ *C*_SP_ alone can be used to approximately extract the preferential-interaction coefficient, *γ*, from Equation (23). Its insertion into Equation (18) then allows the calculation of the hydrodynamic coefficient, *λ*.

## 5. Interpretation of Preferential-Interaction Coefficients

In Section 4, two preferential-interaction coefficients, *γ* and *C*_SP_, were introduced. They appear in the expressions of particle diffusiophoresis (Equation (18)) and cosolute osmotic diffusion (Equation (20)) and are linked to each other by Equation (23). In this section, the behavior of these thermodynamic parameters is discussed by first examining the case of a neutral colloidal particle and then considering the case of charged particles sharing a common ion with an ionic cosolute. According to the Kirkwood–Buff theory [112,113,114,115], we can write the following:

(24) $$C_{\mathrm{SP}}=\;(\Gamma_{\mathrm{SP}}-\Gamma_{\mathrm{SW}})C_{S}$$

where

(25a) $$\Gamma_{\mathrm{SP}}\equiv 4\pi N_{A}\int_{0}^{\infty }[1-g_{\mathrm{SP}}(r)]\;r^{2}dr$$

(25b) $$\Gamma_{\mathrm{SW}}\equiv 4\pi N_{A}\int_{0}^{\infty }[1-g_{\mathrm{SW}}(r)]\;r^{2}dr$$

and *N_A_* is the Avogadros’s number. The functions, *g*_SP_(*r*) and *g*_SW_(*r*), are angularly averaged and normalized distribution functions of cosolute as a function of distance, *r*, from the center of mass of the colloidal particle and solvent molecule, respectively. Note that *g*_SP_(∞) = *g*_SW_(∞) = 1 (bulk value). In the limit of *C*_P_→0, *g*_SP_(*r*) characterizes cosolute radial distribution around one colloidal particle. This distribution is the net result of multiple types of interactions occurring between the colloidal particles, cosolute and solvent molecules. For example, it is shaped not only by the specific binding of solvent and/or cosolute to the particle but also by particle–cosolute excluded-volume interactions. On the other hand, *g*_SW_(*r*) characterizes cosolute–solvent interactions in the binary cosolute–solvent system.

**Figure 5:**
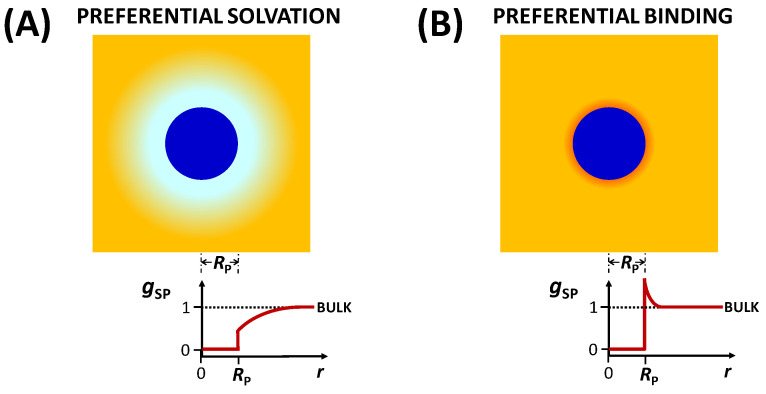
Radial concentration profile of cosolute around a spherical particle of radius *R*_P_ in the case of preferential solvation (**A**) and preferential binding (**B**). Cosolute and solvent molecules are described as a uniform background for simplicity, with the color gradient representing the cosolute gradient. The corresponding plots of the radial distribution function, *g*_SP_(*r*), are also shown. Integration of 1 − *g*_SP_(*r*) between *r* = 0 and *r* = *R*_P_ yields particle volume. Integration of 1 − *g*_SP_(*r*) between *r* = *R*_P_ and *r* = *∞* yields a positive volume in the case of preferential solvation and a negative volume in the case of preferential binding.

The integrals, $\Gamma_{\mathrm{SP}}$ and $\Gamma_{\mathrm{SW}}$, have the units of a molar volume. According to Equation (25a), the steric presence of the colloidal particle itself yields $\Gamma_{\mathrm{SP}}$ = ${\bar{V}}_{P}$. This allows us to appreciate that the magnitude of $\Gamma_{\mathrm{SP}}$ is of the order of ${\bar{V}}_{P}$, while $\Gamma_{\mathrm{SW}}$ is small because its magnitude is comparable with cosolute and solvent molar volumes. Indeed, it can be shown that $\Gamma_{\mathrm{SW}}$ ≈ ${\bar{V}}_{S}$ at low cosolute concentration. To understand the physical meaning of *C*_SP_ and *γ*, we assume that $\Gamma_{\mathrm{SW}}$ << $\Gamma_{\mathrm{SP}}$ in Equation (24) and ${\bar{V}}_{S}$ << ${\bar{V}}_{P}$ in Equation (23). Both G_SP_ and ${\tilde{V}}_{P}$ = ${\bar{V}}_{P}$ are regarded as constant. In this case, we can write the following:

(26a) $$C_{\mathrm{SP}}=\Gamma_{\mathrm{SP}}C_{S}$$

(26b) $$\gamma =\frac{\Gamma_{\mathrm{SP}}-{\bar{V}}_{P}}{1-C_{S}{\bar{V}}_{S}}C_{S}$$

where Equation (26a) shows that *C*_SP_ is directly proportional to cosolute concentration, *C*_S_, with *C*_SP_ = 0 at *C*_S_ = 0. Note that Equation (26b) is obtained from Equation (26a) by using Equation (23). The preferential-interaction coefficient, *g*, is also approximately proportional to *C*_S_ because *C*_S_${\bar{V}}_{S}$ in the denominator of Equation (26b) is usually less than 10%. The proportionality constant, $\Gamma_{\mathrm{SP}}$, represents the net excluded volume experienced by cosolute, with $\Gamma_{\mathrm{SP}}$ = ${\bar{V}}_{P}$ as the reference case in which the steric presence of the colloidal particle is the sole contribution to particle–cosolute interactions. As described in Figure 5, we have $\Gamma_{\mathrm{SP}}$ > ${\bar{V}}_{P}$ when cosolute is depleted in the proximity of the particle (preferential solvation) and $\Gamma_{\mathrm{SP}}$ < ${\bar{V}}_{P}$ when cosolute is enriched in the proximity of the particle (preferential binding).

Clearly, the preferential-interaction coefficient, *γ*, directly quantifies cosolute depletion or enrichment in the particle proximity because *γ* is directly proportional to $\Gamma_{\mathrm{SP}}$ − ${\bar{V}}_{P}$, i.e., *γ* > 0 and *γ* < 0 correspond to preferential solvation and preferential binding, respectively. It is then convenient to rewrite Equation (26b) in the following way [25,79]:

(27) $$\gamma =\nu_{W}\frac{C_{S}}{C_{W}}$$

where we have used *C*_W_${\bar{V}}_{W}$ + *C*_S_${\bar{V}}_{S}$ = 1, with *C*_W_ and ${\bar{V}}_{W}$ being solvent concentration and partial molar volume, respectively. Note that *C*_W_ ≈ 1/${\bar{V}}_{W}$ because *C*_S_${\bar{V}}_{S}$ is small. In Equation (27), we have also introduced the solvent thermodynamic excess, *ν*_W_, through the following equation:

(28) $$\Gamma_{\mathrm{SP}}={\bar{V}}_{P}+\nu_{W}{\bar{V}}_{W}$$

This thermodynamic parameter, which can be approximated as a constant, represents the number of solvent molecules near the colloidal particle in excess with respect to bulk in the case of preferential solvation, and it becomes negative in the case of preferential binding. Although *ν*_W_ increases with particle solvation, it should not be confused [79] with the actual number of solvent molecules bound to the colloidal particles. The solvent thermodynamic excess, *ν*_W_, is introduced by considering the case of a spherical colloidal particle for the sake of simplicity. It is, however, important to note that this thermodynamic formalism is rather general and can be extended to non-spherical particles, including polymer coils. In Section 8, the values of *ν*_W_ extracted for PEG in the presence of osmolytes and salts will be reported.

**Figure 6:**
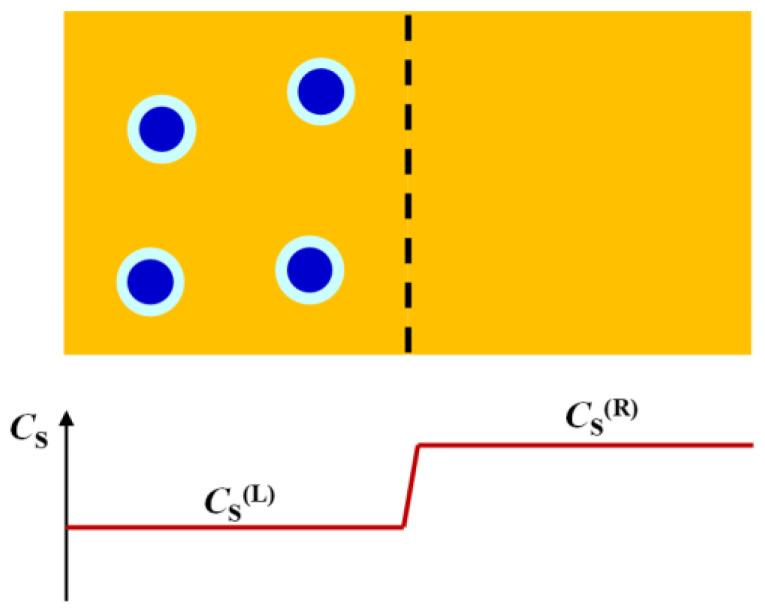
Equilibrium dialysis setup with two compartments containing a ternary particle–cosolute–solvent system (left side) at particle concentration *C*_P_ and cosolute concentration, *C*_S_^(L)^ and a binary cosolute–solvent system (right side) at cosolute concentration, *C*_S_^(R)^. The two compartments are separated by a membrane (dashed line) that is not permeable to particles. Colloidal particles are explicitly shown (blue circles), while cosolute and solvent molecules are described as a uniform background for simplicity. The layer surrounding each particle represents solvent excess near the particle (case of preferential solvation). The bulk cosolute concentration in the left compartment is equal to that of the right compartment, *C*_S_^(R)^. The volume fraction of the left compartment occupied by the particles is (${\bar{V}}_{P}$ + *ν*_W_${\bar{V}}_{W}$)*C*_P_, where ${\bar{V}}_{P}$ characterizes the volume occupied by the particles themselves and *ν*_W_${\bar{V}}_{W}$ their surrounding layers. Since this volume is excluded from the bulk solution, the overall cosolute concentration in the left compartment, *C*_S_^(L)^, is lower than *C*_S_^(R)^. We have *C*_S_^(L)^ = [1 − (${\bar{V}}_{P}$ + *ν*_W_${\bar{V}}_{W}$)*C*_P_]*C*_S_^(R)^. This equilibrium condition can be used to derive *C*_SP_ = −lim*_C_*_P→0_(𝜕*C*_S_/𝜕*C*_P_)*_μ_*_S_ = lim*_C_*_P→0_[(*C*_S_^(L)^ − *C*_S_^(R)^)*/C*_P_] = (${\bar{V}}_{P}$ + *ν*_W_${\bar{V}}_{W}$)*C*_S_, which corresponds to Equations (26a) and (28).

Preferential-interaction coefficients have been extensively investigated in connection to equilibrium dialysis [78,116]. Here, a well-stirred ternary particle–cosolute–solvent solution is in contact with a well-stirred binary cosolute–solvent reservoir through a membrane that is permeable to solvent and cosolute but not to the colloidal particles. At equilibrium, a difference in cosolute concentration between the two solutions (cosolute partitioning) emerges due to preferential solvation or preferential binding. This difference can be explained by observing that the bulk cosolute concentration in the ternary particle–cosolute–solvent compartment is the same as that in the binary cosolute–solvent compartment. This is further described in Figure 6.

**Figure 7:**
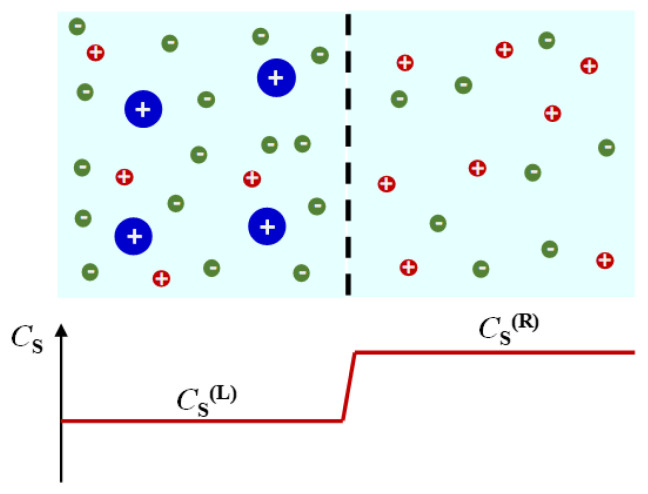
Equilibrium dialysis setup with two compartments containing a ternary particle–cosolute–solvent system (left side) at particle concentration *C*_P_ and cosolute concentration, *C*_S_^(L)^ and a binary cosolute–solvent system (right side) at cosolute concentration, *C*_S_^(R)^, separated by a membrane (dashed line) not permeable to particles. Positively charged colloidal particles and counterions (−) and coions (+) of a 1:1 electrolyte (e.g., NaCl) are explicitly shown, while solvent molecules are described as a uniform background for simplicity. Concentrations of counterion and coion are both equal to *C*_S_^(R)^ in the right compartment, while only the coion concentration is equal to *C*_S_^(L)^ in the left compartment. Here, the counterion concentration is |*Z*_P_|*C*_P_ + *C*_S_^(L)^ > *C*_S_^(L)^ to ensure electroneutrality. At low salt concentration, we have (|*Z*_P_|*C*_P_ + *C*_S_^(L)^) *C*_S_^(L)^ = (*C*_S_^(R)^)^2^. This equilibrium condition can be used to derive *C*_SP_ = −lim*_C_*_P→0_(𝜕*C*_S_/𝜕*C*_P_)*_μ_*_S_ = lim*_C_*_P→0_[(*C*_S_^(L)^ − *C*_S_^(R)^)*/C*_P_] = |*Z*_P_|/2, which leads to the Donnan term in Equations (29) and (30).

Equilibrium dialysis can also be used to determine the expression of *C*_SP_ in the case of a charged colloidal particle in the presence of an ionic cosolute with the membrane permeable to both ions (e.g., Na^+^ and Cl^−^ in NaCl) [72,73]. For simplicity, we shall focus on the case of 1:1 symmetric electrolytes. The ion with a charge that is opposite to that of the particle is denoted as a counterion, while the ion with the same charge is denoted as a coion. This case of equilibrium dialysis corresponds to the well-known *Donnan equilibrium* and has usually been discussed for proteins in the presence of a supporting electrolyte [71,73,81]. To ensure electroneutrality, the concentrations of the two salt ions must be the same in the binary cosolute–solvent compartment. However, there must be an excess of counterions in the ternary particle–cosolute–solvent compartment due to particle charge. If the particle charge is *Z*_P_, the difference in concentration between counterion and coion is |*Z*_P_|*C*_P_. Since the product of the concentrations of the two ions in the ternary compartment must be the same as that in the binary compartment at equilibrium, the excess of counterions in the ternary compartment must be invariably accompanied by a depletion of coions. This leads to a net depression of cosolute concentration in the ternary compartment relative to the binary compartment. Donnan equilibrium is further described in Figure 7. Here, cosolute concentration is assumed to be sufficiently low, so the excluded-volume effect previously illustrated in Figure 6 can be ignored for simplicity.

To take into account the Donnan effect, the expression of the preferential-interaction coefficient, *C*_SP_, is generalized in the following way:

(29) $$C_{\mathrm{SP}}=\frac{\left|Z_{P}\right|}{2}+({\bar{V}}_{P}+\nu_{W}{\bar{V}}_{W})C_{S}$$

where the residual positive value of *C*_SP_ at *C*_S_ = 0 characterizes the Donnan effect, becoming zero for neutral particles. The preferential-interaction coefficient, *γ*, is modified in the same way because there is no difference between *C*_SP_ and *γ* in the limit of *C*_S_→0 (see Equation (23)):

(30) $$\gamma =\frac{\left|Z_{P}\right|}{2}+\nu_{W}\frac{C_{S}}{C_{W}}$$

## 6. Nernst–Planck Equations for Diffusiophoresis and Osmotic Diffusion

For aqueous mixtures containing two (or more) salt components, there exist mathematical expressions straightforwardly linking multicomponent-diffusion coefficients to tracer-diffusion coefficients and the charges of the ions, which are rigorously valid at infinite dilution. These expressions, also known as Nernst–Hartley equations, are derived by applying the electroneutrality conditions to Nernst–Planck equations for single ions [64,71,117]. Clearly, Nernst–Planck equations are also relevant to charged colloidal particles in the presence of a supporting electrolyte. Indeed, there are several multicomponent-diffusion studies on protein–salt–water systems, which have examined the application and validity of Nernst–Planck equations in the case of protein–salt–water systems [37,52,53,117]. In this section, these equations are revisited in order to derive the mathematical expressions of the particle diffusiophoresis coefficient, ${\hat{D}}_{\mathrm{PS}}$, and salt osmotic diffusion coefficient, ${\hat{D}}_{\mathrm{PS}}$, at *C*_S_ = 0. For simplicity, the case of a positively charged particle in the presence of a 1:1 electrolyte will be considered. In this case, salt anions and cations represent particle counterions and coions, respectively. It is also important to bear in mind that we are also operating within the limit of *C*_P_/*C*_S_→0, i.e., we are considering one colloidal particle surrounded by a diluted sea of salt ions.

We start by considering Equation (11) in the limit of *C*_S_→0. We have the following:

(31) $$v_{P}=-D_{P}\left(\nabla \ln C_{P}+2{\hat{D}}_{\mathrm{PS}}\nabla \ln C_{S}\right)$$

According to Nernst–Planck equations, the migration of a charged particle can be induced by gradients of chemical and electrical potentials [71]:

(32) $$v_{P}=-D_{P}\left(\nabla \ln C_{P}+Z_{P}\frac{F\;\nabla \Psi }{RT}\right)$$

where F is Faraday’s constant, ▽ln*C*_P_, which is the same term shown in Equation (31), represents particle chemical-potential gradient in the limit of *C*_P_→0 and ▽Ψ represents the gradient of electrical potential, Ψ. Clearly, Equation (32) is also used to describe particle electrophoresis in response to externally imposed electrical-potential gradients. Migration of salt cations and anions can also be described by Nernst–Plank equations:

(33a) $$-J_{+}=C_{+}D_{+}^{0}\left(\nabla \ln C_{+}+\frac{F\;\nabla \Psi }{RT}\right)$$

(33b) $$-J_{-}=C_{-}D_{-}^{0}\left(\nabla \ln C_{-}-\frac{F\;\nabla \Psi }{RT}\right)$$

where $D_{+}^{0}$ and $D_{-}^{0}$ are the tracer-diffusion coefficients of cation and anion, *J*_+_ and *J*_−_ are the corresponding molar fluxes, and *C*_+_ and *C*_−_ are the corresponding concentrations. Note that the sign change from Equation (33a) to Equation (33b) describes the corresponding change in ion charge.

In a purely diffusive process, there is no external electric field, and the gradient of electrical-potential gradients is internally produced by the gradient of ions (remembering that *C*_P_/*C*_S_→0). In this case, the electrical potential is known as *diffusion potential* and, as it will be shown below, is directly proportional to the difference in mobility between the two ions; note that this potential is also responsible for the liquid junction potential in electrochemical cells. In the absence of external electric fields, the electroneutrality condition holds. This implies that cation and anion must have the same concentration (*C*_+_ = *C*_−_). Moreover, to ensure that electroneutrality is maintained during diffusion, cation and anion must also diffuse at the same diffusion rate even if the two ions have different mobility (*J*_+_ = *J*_−_). The observed salt diffusion coefficient can be determined by first applying *C*_S_ = *C*_+_ = *C*_−_ and *J*_S_ = *J*_+_ = *J*_−_ to Equation (33a,b). We then multiply Equation (33a) by $D_{-}^{0}$ and Equation (33b) by $D_{+}^{0}$. Finally, we add the two resulting equations to eliminate the electric term and obtain the Nernst–Hartley equation of the salt diffusion coefficient, $D_{S}^{0}$, (*D*_S_ in the limit of *C*_S_→0):

(34) $$D_{S}^{0}=\frac{2D_{+}^{0}D_{-}^{0}}{D_{+}^{0}+D_{-}^{0}}$$

According to Equation (34), the salt diffusion coefficient is such that ($D_{S}^{0}$)^−1^ is the average between ($D_{+}^{0}$)^−1^ and ($D_{-}^{0}$)^−1^. The expression of ▽Ψ is similarly obtained by taking the difference between Equation (33a) and Equation (33b). This yields the following:

(35) $$\frac{F\;\nabla \Psi }{RT}=\frac{D_{-}^{0}-D_{+}^{0}}{D_{-}^{0}+D_{+}^{0}}\;\nabla \;\ln C_{S}=\Delta \tau \;\;\nabla \ln C_{S}$$

where Δ*τ* = *τ*_−_ − *τ*_+_, *τ*_+_ = $D_{+}^{0}$/($D_{+}^{0}$ + $D_{-}^{0}$) and *τ*_−_ = $D_{-}^{0}$/($D_{+}^{0}$ + $D_{-}^{0}$) are cation and anion *transference numbers*, respectively, with *τ*_−_ = 1 − *τ*_+_ [105]. Tracer-diffusion coefficients are available [118] for several ions in water at 25 °C and can be used to evaluate Δ*τ*. For example, Δ*τ* = 0.21 for NaCl and Δ*τ* = 0.02 for KCl. Each charged species, including the colloidal particle, will experience this internal electric field. If we insert Equation (35) into Equation (32) and compare it with Equation (31), we obtain the following:

(36) $${\hat{D}}_{\mathrm{PS}}=\frac{\left|Z_{P}\right|}{2}\Delta \tau$$

where we have replaced Z_P_ with |Z_P_| so that we can readily compare Equation (36) with the Donnan term, |*Z*_P_|/2 (see Equations (29) and (30) with *C*_S_ = 0). For negatively charged particles, *τ*_+_ and *τ*_−_ must be inverted. Alternatively, we can directly apply Equation (36) to negative particles provided that we remember that Δ*τ* = *τ*_C_ − *τ*_S_ is defined as the difference between the transference number of counterion (*τ*_C_) and coion (*τ*_S_). Since particle diffusiophoresis is induced by an internal electric field, Equation (36) characterizes the mechanism of electrophoretic diffusiophoresis in the limit of *C*_S_→0. Nearly all studies on diffusiophoresis are based on mathematical expressions of diffusiophoresis coefficients that are generalizations of Equation (36) [12,13,16,19,20,72].

The corresponding expression of the hydrodynamic coefficient, *λ*, can be obtained from Equation (18). If we subtract Equation (36) to *g* = |*Z*_P_|/2, we obtain the following:

(37) $$\lambda =|Z_{P}|\;\tau_{S}$$

where |*Z*_P_| represents the number of counterions and *τ*_S_ is the transference number of the coion (*τ*_+_ if *Z*_P_ > 0 and *τ*_−_ if *Z*_P_ < 0).

We can readily obtain also the expression of the salt osmotic diffusion coefficient. If we insert *C*_SP_ = |*Z*_P_|/2 and Equation (37) for *λ* in Equation (20), we obtain the following:

(38) $${\hat{D}}_{\mathrm{SP}}=\frac{\left|Z_{P}\right|}{2}(1-2\alpha \tau_{S})$$

where $D_{S}^{0}$ in *α* = $D_{P}$/$D_{S}^{0}$ is evaluated using Equation (34). Note that ${\hat{D}}_{\mathrm{SP}}$ ≈ |*Z*_P_|/2 is a suitable approximation. Since salt osmotic diffusion is approximately described by the Donnan term, |*Z*_P_|/2, Equation (38) characterizes the mechanism of Donnan diffusion. The applicability and limitations of Equations (36)–(38) will be discussed in Section 11.

## 7. Extraction of ${\hat{D}}_{\mathrm{PS}}$(*C*_S_) and ${\hat{D}}_{\mathrm{SP}}$(*C*_S_) from Ternary Diffusion Coefficients

As previously mentioned, multicomponent-diffusion coefficients in the volume-fixed reference, $D_{i,j}^{V}$, can be determined using Taylor’s dispersion or interferometric methods. The experimental results that are discussed in the following sections were obtained using the Gosting diffusiometer operating in the Rayleigh interferometry method at 25 °C [37,62,119,120,121,122]. This instrument, which is located at Texas Christian University in Fort Worth, Texas (USA), yields diffusion coefficients at the highest precision [37,123,124]. This instrument was initially built by Louis J. Gosting at the University of Winsconsin-Madison and then optimized by Donald G. Miller at Lawrence Livermore National Laboratory and John G. Albright at Texas Christian University [125]. Although experiments on the Gosting diffusiometer typically require specialized user training and may be time consuming, the precision of the Rayleigh interferometry method was found to be particularly important for successfully characterizing the dependence of diffusiophoresis and osmotic diffusion coefficients (${\hat{D}}_{\mathrm{PS}}$ and ${\hat{D}}_{\mathrm{SP}}$) on cosolute concentration, *C*_S_ [23,25,72,74,92,94,95,126].

**Figure 8:**
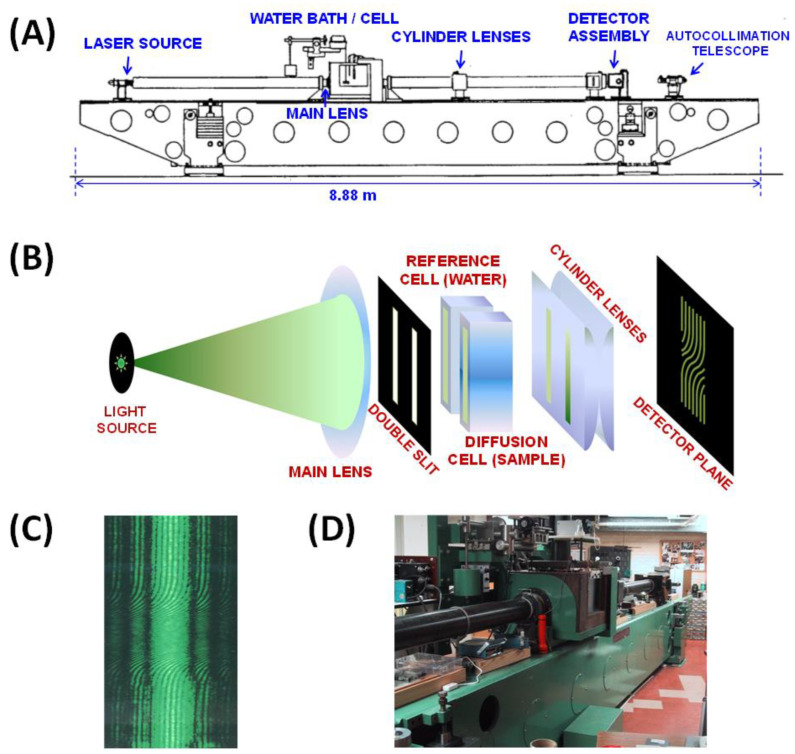
(**A**) Schematic diagram showing the main components of the Gosting diffusiometer. (**B**) Schematic diagram showing the optical components employed for generating a Rayleigh interferometric pattern from two solutions vertically interfaced inside a diffusion cell. (**C**) Picture of a Rayleigh interferometric pattern. (**D**) Picture showing the Gosting diffusiometer at Texas Christian University.

As illustrated in Figure 8, the Gosting diffusiometer consists of a ≈9 m optical bench (5000 kg) with vibration isolation and several components [120]. The light source for generating the Rayleigh interference pattern is a He-Ne laser (543.5 nm, 5 mW). The lens components are the main lens (two-element air-spaced achromat, focal length 145 cm) and the cylinder lens (two plano-convex lenses, focal length 68 cm). A cell holder is located between these two lens components inside a water bath. The temperature of the bath was regulated at 25.00 °C with a model PTC-41 Tronac temperature controller to a precision of (0.001 °C). The cell holder has the function of supporting a Tiselius cell (where macroscopic-gradient diffusion occurs) and a mask, which consists of a double window. Here, the laser beam is split into two parts: one going through the diffusion channel of the Tiselius cell and one passing through the water bath (reference channel). The cylinder lens focuses the diffusion channel at the detector, where the Rayleigh interference pattern is observed [62]. Data from the Rayleigh interference patterns are collected with a linear CCD array (6000 pixels, 10 µm × 10 µm pixels) mounted vertically on a precision stage [37,119]. The stage with this vertical array is stepped horizontally through the 2D interference pattern to collect the data necessary to calculate the diffusion coefficients. The magnification factor is measured using a precision ruled quartz scale (100 lines/cm, accuracy 0.25 µm; Photo Sciences Inc., Torrance, CA, USA).

A typical diffusion experiment begins by preparing a sharp boundary between two solutions of different solute concentrations in the vertical diffusion channel (cell) located inside the thermostated water bath. The measured ternary diffusion coefficients correspond to the average concentrations of the two interfaced solutions. Rayleigh interferometric fringes shift horizontally as the refractive index inside the diffusion channel changes along the channel’s vertical position. This shift is directly proportional to the medium refractive index. The total number of fringes is directly proportional to the difference in refractive index between the two initial solutions and the channel width and inversely proportional to the laser wavelength. The differences in concentrations between the two interfaced solutions are typically chosen to obtain ≈50 fringes. Refractive-index profiles are typically determined at 50 different values of time during the course of each experiment. These are then rearranged as anti-symmetric and normalized sigmoidal functions, which are theoretically described as linear combinations of two error functions. A minimum of two experiments is required to determine the four diffusion coefficients at a given set of average concentrations. These two experiments must have different combinations of solute concentration differences across the diffusion boundary. To verify reproducibility, two other duplicate experiments are performed. The four ternary diffusion coefficients in the volume-fixed reference frame are then extracted by applying a method of the non-linear least squares to all normalized refractive-index profiles [127,128].

The reduced particle diffusiophoresis, ${\hat{D}}_{\mathrm{PS}}$(*C*_S_), is obtained from the cross-term, $D_{\mathrm{PS}}^{V}$, by using the following [25]:

(39) $${\hat{D}}_{\mathrm{PS}}=\left(\frac{1}{D_{P}}\underset{C_{P}\to 0}{\lim}\frac{D_{\mathrm{PS}}^{V}}{C_{P}}+\frac{{\bar{V}}_{S}}{\alpha }\right)\frac{C_{S}}{\nu_{S}y_{S}}$$

where *C*_S_/(*ν*_S_*y*_S_) represents the conversion factor from ∇*μ*_S_/*RT* to ∇*C*_S_ (see Equation (10)) and the term ${\bar{V}}_{S}$/*α* represents a small correction (from Equation (9c)), accounting for the change from solvent- to volume-fixed reference frame. Cosolute partial molar volumes, ${\bar{V}}_{S}$, and thermodynamic factors, *y*_S_, are typically available from the literature.

The reduced cosolute osmotic diffusion coefficient, ${\hat{D}}_{\mathrm{SP}}$(*C*_S_), is obtained from the cross-term, $D_{\mathrm{SP}}^{V}$, by using the following [25]:

(40) $${\hat{D}}_{\mathrm{SP}}\equiv \underset{C_{P}\to 0}{\lim}\;\;\frac{D_{\mathrm{SP}}^{V}}{D_{\mathrm{SS}}^{V}}+\alpha \frac{C_{S}{\bar{V}}_{P}}{1-C_{S}{\bar{V}}_{S}}$$

where the term *αC*_S_${\bar{V}}_{P}$/(1 − *C*_S_${\bar{V}}_{S}$) also represents a small correction (from Equation (9c)), accounting for the change from solvent- to volume-fixed reference frame.

According to Equations (39) and (40), ${\hat{D}}_{\mathrm{PS}}$ and ${\hat{D}}_{\mathrm{SP}}$ should be extracted by measuring $D_{\mathrm{PS}}^{V}$/*C*_P_, $D_{\mathrm{SP}}^{V}$/$D_{\mathrm{SS}}^{V}$ as a function of *C*_P_ at a given *C*_S_ and then extrapolating these two quantities to *C*_P_→0. However, we find that this extrapolation is not needed as long as the particle concentrations are less than ≈1% (*w/w*) [23,72]. In other words, $D_{\mathrm{PS}}^{V}$/*C*_P_, $D_{\mathrm{SP}}^{V}$/$D_{\mathrm{SS}}^{V}$ are found to be independent of *C*_P_ within the experimental error at low particle concentration. Furthermore, measurements of cross-term diffusion coefficients at very low particle concentrations yield relatively large errors. Specifically, the relative error of $D_{\mathrm{PS}}^{V}$/*C*_P_ increases as *C*_P_ decreases because $D_{\mathrm{PS}}^{V}$ is directly proportional to *C*_P_. Moreover, the error on the other cross-term, $D_{\mathrm{SP}}^{V}$, also increases as *C*_P_ decreases. This occurs because the difference in particle concentration between the two solutions in a diffusion experiment is also limited by *C*_P_.

We now discuss why thermodynamic non-ideality associated with particle–particle interactions is not expected to significantly affect $D_{\mathrm{PS}}^{V}$/*C*_P_ and $D_{\mathrm{SP}}^{V}$/$D_{\mathrm{SS}}^{V}$. The thermodynamic factor, *C*_P_*μ*_PP_/*RT*, characterizes this non-ideality effect, with *C*_P_*μ*_PP_/*RT*→1 in the limit of *C*_P_→0. It is important to note that this parameter can show appreciable dependence on *C*_P_ even at low particle concentrations (≈1%), especially in the proximity of a spinodal boundary [23]. Inspection of Equation (5a–d) shows that only *D*_PP_ and *D*_SP_ depend on *μ*_PP_. This implies that $D_{\mathrm{PS}}^{V}$/*C*_P_ is independent of *C*_P_*μ*_PP_/*RT*. On the other hand, the cross-term, *D*_SP_, does depend on *μ*_PP_. However, its contribution to ${\hat{D}}_{\mathrm{SP}}$ is limited to the *al* term in Equation (20). Since this is small compared to *C*_SP_, we deduce that the effect of *C*_P_*μ*_PP_/*RT* on $D_{\mathrm{SP}}^{V}$/$D_{\mathrm{SS}}^{V}$ is also negligible.

The values of *D*_P_ as a function of *C*_S_ are also needed in Equation (39). According to Equation (9a), this could be extracted, at any given *C*_S_, by measuring $D_{\mathrm{PP}}^{V}$ as a function of *C*_P_ and then extrapolating to *C*_P_→0. However, a more rapid and versatile strategy to determine *D*_P_ is by extrapolation of DLS diffusion coefficients as a function of *C*_P_ [23].

## 8. The PEG–Osmolyte–Water and PEG–Salt–Water Systems

In this section, representative ${\hat{D}}_{\mathrm{PS}}$(*C*_S_) and ${\hat{D}}_{\mathrm{SP}}$(*C*_S_) data extracted from ternary diffusion coefficients at 25 °C using Rayleigh interferometry on aqueous PEG (nominal molecular weight, 20 kg/mol) in the presence of osmolytes and salts will be discussed [25,92,94]. PEG is a nonionic hydrophilic polymer found in many aqueous formulations relevant to pharmaceutical and biotechnological applications [129,130,131]. Furthermore, an important class of water-soluble colloidal particles is represented by neutral particles whose interfacial properties are modified or governed by PEG. Indeed, this polymer has been employed to coat the surface of inorganic nanoparticles [132,133], proteins [134], micelles [135] and vesicles [136,137]. Since diffusiophoresis is mostly an interfacial phenomenon, understanding diffusiophoresis of PEG coils is of fundamental importance for understanding diffusiophoresis of PEG-based colloidal particles [23,74].

We start by considering the effect of three osmolytes, trimethyl-N-oxide (TMAO), diethylene glycol (DEG) and urea, on PEG diffusiophoresis. Osmolytes are small organic compounds that are neutral and water soluble. They have been extensively investigated in connection with proteins because they are known to significantly affect the thermodynamic stability of the protein native state through the mechanism of preferential hydration [79,138,139]. The same type of mechanism is observed also in the case of hydrophilic macromolecules such as PEG [25].

**Figure 9:**
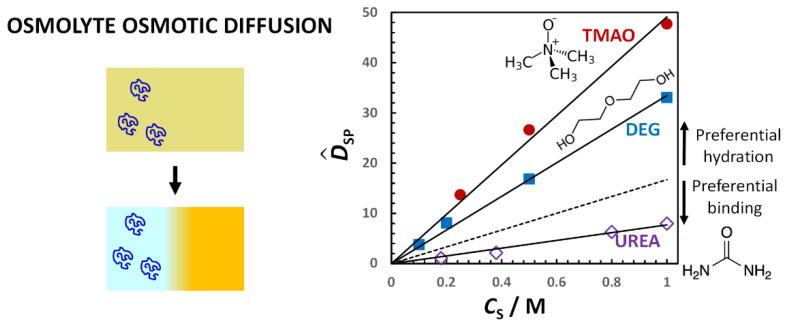
Osmotic diffusion of osmolyte occurs from high to low concentration of PEG coils. Osmolyte osmotic diffusion coefficient, ${\hat{D}}_{\mathrm{SP}}$, as a function of osmolyte concentration, *C*_S_, in the TMAO (●), DEG (■) and urea (◇) cases [25]. Dashed line has zero intercept and slope equal to ${\bar{V}}_{P}$.

Due to its more direct connection with the preferential-interaction coefficient, it is convenient to examine osmolyte osmotic diffusion prior to PEG diffusiophoresis. In Figure 9, the osmolyte (TMAO, DEG and urea) osmotic diffusion coefficient, ${\hat{D}}_{\mathrm{SP}}$, as a function of osmolyte concentration, *C*_S_, is shown. As we can see, ${\hat{D}}_{\mathrm{SP}}$ data are positive, thereby implying that osmolyte diffuses from high to low PEG concentration. We can also see that ${\hat{D}}_{\mathrm{SP}}$ linearly increases with *C*_S_ starting from ${\hat{D}}_{\mathrm{SP}}$(0) = 0, consistent with ${\hat{D}}_{\mathrm{SP}}$ ≈ *C*_SP_ = (${\bar{V}}_{P}$ + *ν*_W_${\bar{V}}_{W}$)·*C*_S_ (see Equation (29) with *Z*_P_ = 0). For comparison, the reference line, ${\bar{V}}_{P}$*C*_S_ (with ${\bar{V}}_{P}$ = 16.7 dm^3^·mol^−1^) [92], is included in the same figure. Positive deviations from this line correspond to positive values of water thermodynamic excess, *ν*_W_. We can then deduce that PEG is preferentially hydrated in the presence of TMAO and DEG. Indeed, the application of Equations (18), (20) and (23) to ${\hat{D}}_{\mathrm{PS}}$(*C*_S_) and ${\hat{D}}_{\mathrm{SP}}$(*C*_S_) data, which confirms that ${\hat{D}}_{\mathrm{SP}}$ ≈ *C*_SP_ is a suitable approximation, rigorously yields *γ*(*C*_S_). We can then write *γ* = *ν*_W_*C*_S_/*C*_W_ and determine the thermodynamic excess of water molecules near PEG. This is higher for TMAO (*ν*_W_ = 1870; 4.1 per ethoxy group) than for DEG (*ν*_W_ = 970; 2.1 per ethoxy group). On the other hand, urea is preferentially binding to PEG because ${\hat{D}}_{\mathrm{SP}}$ data are below the reference line. In this case, the water thermodynamic excess is negative (*ν*_W_ = −520; −1.1 per ethoxy group). It is interesting to observe that the effect of osmolytes on PEG follows the same type of trend observed in the case of protein. Indeed, the TMAO preferential-hydration effect on proteins is known to stabilize the protein native state. In contrast, urea preferentially binds to peptide chains. This is known to destabilize the protein native state, leading to unfolding [138].

**Figure 10:**
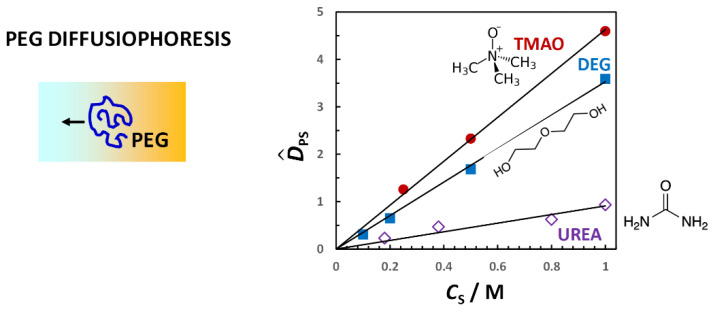
Diffusiophoresis of PEG occurs from high to low osmolyte concentration. PEG diffusiophoresis coefficient, ${\hat{D}}_{\mathrm{PS}}$, as a function of osmolyte concentration, *C*_S_, in the TMAO (●), DEG (■) and urea (◇) cases [25]. Diagram on the right describes.

In Figure 10, the PEG diffusiophoresis coefficient, ${\hat{D}}_{\mathrm{PS}}$, as a function of osmolyte concentration, *C*_S_, is shown. As we can see, ${\hat{D}}_{\mathrm{PS}}$ data are also positive, thereby implying that PEG diffusiophoresis occurs from high to low osmolyte concentration in all three cases. Moreover, as in the case of osmotic diffusion data, ${\hat{D}}_{\mathrm{PS}}$ linearly increases with *C*_S_ starting from ${\hat{D}}_{\mathrm{PS}}$(0) = 0. The slope trend of diffusiophoresis data is the same as that of osmotic diffusion data in Figure 9, with PEG diffusiophoresis being the largest in the TMAO case. In the urea case, ${\hat{D}}_{\mathrm{PS}}$ values are small compared to those observed for the other two osmolytes. Interestingly, although PEG prefers to interact more with urea than water, PEG diffusiophoresis still occurs from high to low urea concentration.

According to Equation (18), ${\hat{D}}_{\mathrm{PS}}$ is the difference between the preferential-interaction coefficient, *γ*, and the hydrodynamic coefficient, *λ*. Since both ${\hat{D}}_{\mathrm{PS}}$ and *γ* are directly proportional to *C*_S_, the ratio, ${\hat{D}}_{\mathrm{PS}}$/*γ*, is a constant with ${\hat{D}}_{\mathrm{PS}}$/*γ* = 1 when *λ* = 0. In general, the value of this ratio can be used to assess the significance of the hydrodynamic coefficient, *λ*. Examination of our experimental data using Equations (18), (20) and (23) yields ${\hat{D}}_{\mathrm{PS}}$/*γ* = 0.14, 0.19 and −0.10 in the TMAO, DEG and urea case, respectively [25]. This implies that *λ* significantly contributes to the value of ${\hat{D}}_{\mathrm{PS}}$, with *λ*/*γ* = 0.86, 0.81 and 1.10, respectively. We can also appreciate that *l* has the same sign as *γ* (e.g., positive in the presence of preferential hydration) and is directly proportional to *C*_S_, with *λ*(0) = 0.

**Figure 11:**
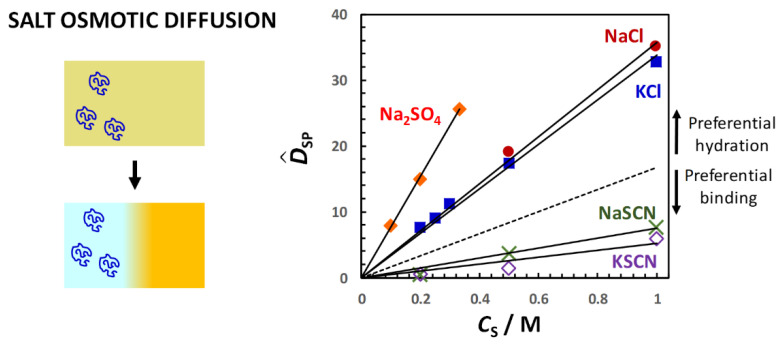
Osmotic diffusion of salt occurs from high to low concentrations of PEG coils. Salt osmotic diffusion coefficient, ${\hat{D}}_{\mathrm{SP}}$, as a function of salt concentration, *C*_S_, in the Na_2_SO_4_ (◆), NaCl (●), KCl (■), NaSCN (✕) and KSCN (◇) cases [94,95]. Dashed line has zero intercept and slope equal to ${\bar{V}}_{P}$.

Similar results are obtained for PEG in the presence of salts [92,94]. In Figure 11, the salt osmotic diffusion coefficient, ${\hat{D}}_{\mathrm{SP}}$, as a function of salt concentration, *C*_S_, is shown. As previously mentioned, the salts are NaCl, KCl, NaSCN, KSCN and Na_2_SO_4_. All ${\hat{D}}_{\mathrm{SP}}$ data are positive as in the osmolyte case, with ${\hat{D}}_{\mathrm{SP}}$(0) = 0, as expected for neutral macromolecules. As in the case of osmolytes, the increase in ${\hat{D}}_{\mathrm{SP}}$ with *C*_S_ is consistent with ${\hat{D}}_{\mathrm{SP}}$ ≈ *C*_SP_ = (${\bar{V}}_{P}$ + *ν*_W_${\bar{V}}_{W}$)*C*_S_. Comparison with the reference line, ${\bar{V}}_{P}$*C*_S_, shows that PEG is preferentially hydrated in the presence of NaCl, KCl and Na_2_SO_4_. Combination of ${\hat{D}}_{\mathrm{PS}}$(*C*_S_) and ${\hat{D}}_{\mathrm{SP}}$(*C*_S_) data yield a water thermodynamic excess that is significantly higher for Na_2_SO_4_ (*ν*_W_ = 3500; 7.7 per ethoxy group) than for NaCl (*ν*_W_ = 1100; 2.4 per ethoxy group) and KCl (*ν*_W_ = 990; 2.2 per ethoxy group) [92]. On the other hand, ${\hat{D}}_{\mathrm{SP}}$(*C*_S_) data exhibit negative deviations from the reference line in the case of thiocyanate salts, thereby implying preferential binding with negative *n*_W_ values for NaSCN (*ν*_W_ = −550; −1.2 per ethoxy group) and KSCN (*ν*_W_ = −690; −1.5 per ethoxy group) [94]. This is attributed to the preferential binding of thiocyanate anions on the PEG polymer chain. Clearly, changing anion nature has an effect on salt osmotic diffusion that is large compared to the effect observed by changing cation from sodium to potassium.

The observed effect of salts on PEG also follows the same type of trend observed in the case of proteins, with sulfate salts known to stabilize the protein native state while thiocyanate salts favor protein unfolding [78]. Moreover, inorganic ions have been ranked according to their effectiveness in precipitating proteins and synthetic polymers (salting-out strength), leading to the well-known Hofmeister series [140]. In this series, the sulfate anion displays a great salting-out strength, whereas chloride is approximately at the midpoint of the Hofmeister series, separating salting-out from salting-in anions such as thiocyanate, which increase the solubility of macromolecules in water. Compared to anions, the Hofmeister series for cations is significantly less pronounced, and the cation ranking can depend on the chemical nature of the macromolecule investigated, with sodium and potassium cations exhibiting similar salting-out strength. Thus, salt osmotic diffusion data are consistent with the Hofmeister series.

**Figure 12:**
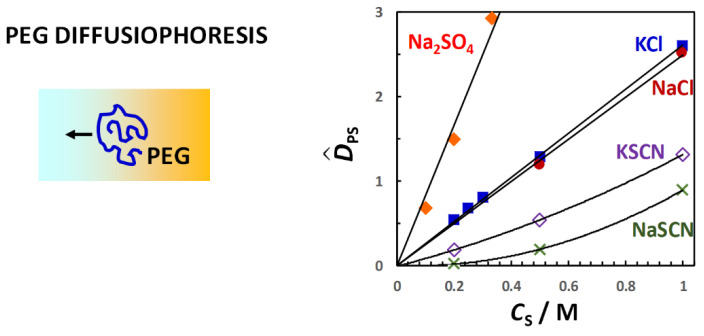
Diffusiophoresis of PEG occurs from high to low salt concentration. PEG diffusiophoresis coefficient, ${\hat{D}}_{\mathrm{PS}}$, as a function of salt concentration, *C*_S_, in the Na_2_SO_4_ (◆), NaCl (●), KCl (■), NaSCN (✕) and KSCN (◇) cases [94,95].

In Figure 12, the PEG diffusiophoresis coefficient, ${\hat{D}}_{\mathrm{PS}}$, as a function of salt concentration, *C*_S_, is shown, with ${\hat{D}}_{\mathrm{PS}}$(0) = 0. As we can see, ${\hat{D}}_{\mathrm{PS}}$ data are positive, thereby implying that PEG diffusiophoresis occurs from high to low salt concentrations. Except for thiocyanate salts, ${\hat{D}}_{\mathrm{PS}}$ linearly increases with *C*_S_. This is the same behavior previously shown for PEG in the presence of osmolytes. Moreover, the slope trend of diffusiophoresis data is the same as that of osmotic diffusion data in Figure 9, with PEG diffusiophoresis being the largest in the Na_2_SO_4_ case. In other words, ${\hat{D}}_{\mathrm{PS}}$ appears to be approximately proportional to *γ*. As in the osmolyte case, ${\hat{D}}_{\mathrm{PS}}$(*C*_S_) and ${\hat{D}}_{\mathrm{PS}}$(*C*_S_) data can be combined to determine ${\hat{D}}_{\mathrm{PS}}$/*γ*. We obtain ${\hat{D}}_{\mathrm{PS}}$/*γ* = 0.14, 0.13 and 0.12 in the NaCl, KCl and Na_2_SO_4_ cases, respectively. This implies that *l* significantly contributes to the observed value of ${\hat{D}}_{\mathrm{PS}}$ also in the salt cases, with *λ*/*γ* = 0.86, 0.87 and 0.88, respectively [92]. Moreover, even if the value increases more than three-fold when going from NaCl and KCl to Na_2_SO_4_, the value of ${\hat{D}}_{\mathrm{PS}}$/*γ* is approximately the same not only among these salts but also when comparing salts and osmolytes.

For the two thiocyanate cases, ${\hat{D}}_{\mathrm{PS}}$ values are relatively small. However, ${\hat{D}}_{\mathrm{PS}}$(*C*_S_) is appreciably larger for KSCN than for NaSCN, especially at low salt concentrations. The difference between the two salt cases can be explained by considering the actual binding of thiocyanate anions to PEG [94]. This process induces a negative charge on the polymer chain, making PEG feel the diffusion potential generated by the salt gradient. The contribution of electrophoretic diffusiophoresis to total diffusiophoresis has been evaluated using Equation (36) and removed from experimental values of ${\hat{D}}_{\mathrm{PS}}$(*C*_S_) [94]. However, detailed calculation of electrophoretic diffusiophoresis will not be discussed here. Instead, for simplicity, we shall just focus on the difference, Δ*τ*, between counterion (Na^+^ or K ^+^) and coion (SCN^−^) transference numbers, which is Δ*τ* = −0.14 for NaSCN and Δ*τ* = +0.05 for KSCN [104,118,141]. According to Equation (36), NaSCN causes a negative electrophoretic diffusiophoresis, while KSCN causes a small positive electrophoretic diffusiophoresis. Thus, the difference between the two salt cases in Figure 11 can be explained by considering that electrophoretic diffusiophoresis reduces the value of ${\hat{D}}_{\mathrm{PS}}$ in the NaSCN case relative to the KSCN case.

In summary, experimental values of ${\hat{D}}_{\mathrm{PS}}$ and ${\hat{D}}_{\mathrm{SP}}$ as a function of cosolute concentration, *C*_S_, show that both ${\hat{D}}_{\mathrm{PS}}$(*C*_S_) and ${\hat{D}}_{\mathrm{SP}}$(*C*_S_) are positive quantities for neutral PEG. In other words, PEG diffusiophoresis (cosolute osmotic diffusion) occurs from high to low cosolute (PEG) concentrations. Furthermore, these two coefficients are proportional to *C*_S,_ with ${\hat{D}}_{\mathrm{PS}}$(0) = ${\hat{D}}_{\mathrm{SP}}$(0) = 0. It was also verified that ${\hat{D}}_{\mathrm{SP}}$ ≈ *C*_SP_ is approximately a thermodynamic quantity directly related to the preferential-interaction coefficient, *γ* = *ν*_W_*C*_S_/*C*_W_. The thermodynamic excess of water molecules near PEG coils, *ν*_W_, was determined for selected osmolytes and salts and was found to follow the same trend observed for proteins. In the presence of preferential hydration (*ν*_W_ > 0), PEG diffusiophoresis, which is approximately proportional to *ν*_W_, occurs in the direction in which PEG chemical potential is lowered as expected. According to data analysis, the ratios, *λ*/*γ* and ${\hat{D}}_{\mathrm{PS}}$/*γ* = 1 − *λ*/*γ*, can be regarded as constants because the hydrodynamic coefficient, *λ*, is also proportional to *C*_S_, with *λ*(0) = 0. In these cosolute cases (TMAO, DEG, sulfate and chloride salts), it was found that ${\hat{D}}_{\mathrm{PS}}$/*γ* = 0.10–0.20. This implies that ${\hat{D}}_{\mathrm{PS}}$ ≈ *γ* is *not* a suitable approximation. In other words, ${\hat{D}}_{\mathrm{PS}}$ cannot be approximated as a thermodynamic quantity as in the case of ${\hat{D}}_{\mathrm{SP}}$. Interestingly, values of *λ*/*γ* for different cosolutes tend to cluster together even if *ν*_W_ changes significantly. Another interesting result is that diffusiophoresis remains positive in the presence of cosolutes that preferentially bind to PEG, i.e., with negative *ν*_W_ (thiocyanates and urea). In other words, PEG diffusiophoresis still occurs from high to low cosolute concentrations even if PEG chemical potential tends to increase in this direction. However, the magnitude of ${\hat{D}}_{\mathrm{PS}}$ is relatively small compared to the cases in which *ν*_W_ is positive. In the following section, a model that can explain these experimental findings will be introduced.

## 9. Local-Domain Model

The observed behavior of diffusiophoresis can be examined by reconsidering a local-domain model, which was previously introduced to describe preferential-interaction coefficients [25,81]. The case of a spherical colloidal particle will be considered for simplicity. However, it is important to remark that relevant equations do not rely on particle shape and are also valid for polymer coils such as PEG. According to this model, the region surrounding a colloidal particle has a composition that is different from bulk, consistent with Kirkwood–Buff theory (see Figure 5). This region, which is assumed to have a fictitious boundary, is denoted as a local domain. The remaining solution, which is a binary cosolute–solvent reservoir, represents the bulk domain. The composition of the local domain can be linked to the composition of the bulk domain by formally introducing a two-phase partitioning coefficient, *K*, with *N*_S_/*N*_W_ = *K C*_S_/*C*_W_, where *N*_S_ and *N*_W_ are the cosolute and water number of molecules in the local domain. For salts, *N*_S_ should be taken as the average of cation and anion numbers in the local domain. The absence of preferential interaction corresponds to *K* = 1. Preferential hydration corresponds to *K* < 1, with full depletion of cosolute achieved in the local domain when *K*→0. Cosolute preferential binding corresponds to *K* > 1, with full depletion of solvent achieved when *K*→∞. Within the framework of this model, the solvent thermodynamic excess is *ν*_W_ = *N*_W_(1 − *K*), with *ν*_W_ = *N*_W_ corresponding to the full depletion of cosolute. It is impossible to determine *N*_W_ and *K* separately as *ν*_W_ is the only experimentally accessible parameter. Furthermore, this model does not explicitly consider the actual binding of cosolute or solvent to the particle and excluded-volume interactions. For example, the concentration of cosolute molecules near the particle surface may be different from bulk because their size is different from that of solvent molecules [113]. If cosolute is larger than solvent, a depletion of cosolute occurs in the local domain without the need to consider other physical or chemical interactions. Nonetheless, as will be shown below, this model provides a useful conceptual picture for understanding how preferential hydration affects diffusiophoresis.

In the case of diffusiophoresis, it becomes important to distinguish between molecules (or ions) that are actually bound to the colloidal particle and those that are not bound but still contribute to the local domain [25]. Bound molecules move together with the particle during transport processes in liquids. To distinguish between these two cases, the local domain is split into an inner local domain (I), in which molecules move together with the particle, and an outer local domain (II), enclosing molecules that are still thermodynamically affected by the particle but moving together with bulk fluid. Note that a well-defined separation into two domains is a model assumption. Indeed, it is expected that there are many fluid molecules moving with intermediate velocities, consistent with hydrodynamic models. Furthermore, the division into an inner domain with bound molecules and an outer domain with non-bound molecules is certainly reasonable but not general. For example, excluded-volume interactions invariably start at the particle surface, but they are formally attributed to the outer domain [142].

**Figure 13:**
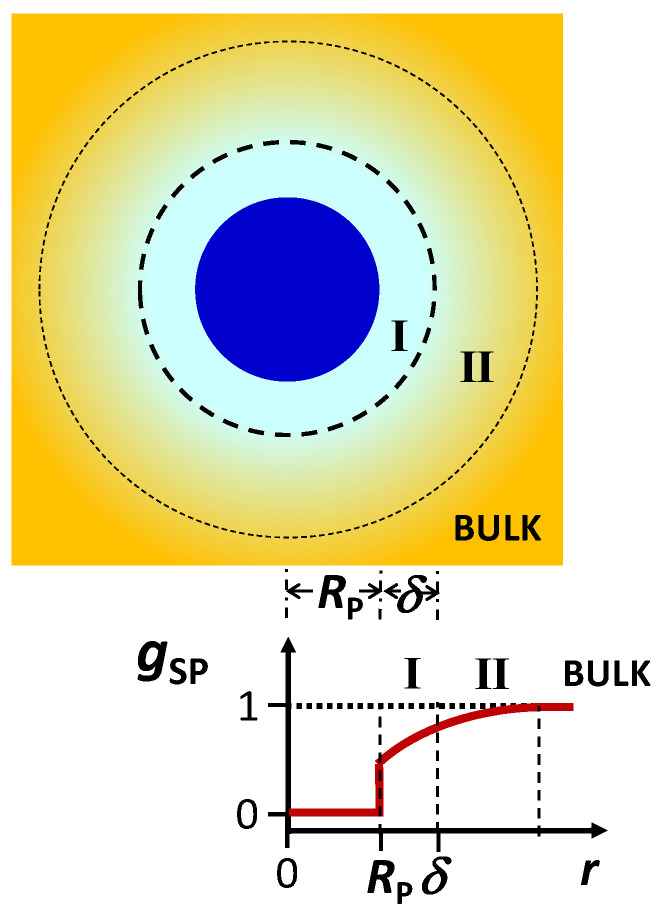
Radial concentration profile of cosolute around a spherical particle of radius *R*_P_ in the case of preferential hydration. The outer dashed circle separates the local domain from the bulk domain. The inner dashed circle separates the inner domain (I) from the outer domain (II). Cosolute and solvent molecules are described as a uniform background for simplicity, with the color gradient representing the cosolute gradient. The corresponding plot of the radial distribution function, *g*_SP_(*r*), is also shown, with *r* = *δ* representing the location of the boundary between the inner and outer domains.

The splitting of the local domain is illustrated in Figure 13 for a spherical particle. Cosolute and solvent in the inner and outer local domains are in chemical equilibrium with the binary cosolute–solvent bulk domain. As discussed below, it is only the outer domain that is responsible for diffusiophoresis.

As in the case of the original local-domain model, the composition of the inner (I) and outer (II) local domains are linked to the composition of the bulk domain by formally introducing partitioning coefficients, *K*^(I)^ and *K*^(II)^, with $N_{S}^{\left(I\right)}$/$N_{W}^{\left(I\right)}$ = *K*^(I)^*C*_S_/*C*_W_ and $N_{S}^{\left(\mathrm{II}\right)}$/$N_{W}^{\left(\mathrm{II}\right)}$ = *K*^(II)^*C*_S_/*C*_W_, where $N_{S}^{\left(I\right)}$ and $N_{W}^{\left(I\right)}$, and $N_{S}^{\left(\mathrm{II}\right)}$ and $N_{W}^{\left(\mathrm{II}\right)}$ are the cosolute and water number of molecules in domains I and II, respectively. The absence of preferential interaction in domains (I) and (II) corresponds to *K*^(I)^ = 1 and *K*^(II)^ = 1, respectively. Preferential hydration in the inner and outer domain corresponds to *K*^(I)^ < 1 and *K*^(II)^ < 1, with full depletion of cosolute achieved in the domains in the limits of *K*^(I)^→0 and *K*^(II)^→0. On the other hand, cosolute preferential binding corresponds to *K*^(I)^ > 1 and *K*^(II)^ > 1, with full depletion of solvent achieved in the limits of *K*^(I)^→∞ and *K*^(II)^→∞. It is expected that |1 − *K*^(II)^| < |1 − *K*^(I)^| as interactions with the particle are relatively weak in the outer local domain. Although |1 − *K*^(II)^| and |1 − *K*^(I)^| may significantly depend on cosolute nature, it is expected that the ratio, |1 − *K*^(II)^|/|1 − *K*^(I)^|, which describes the relative change in interaction strength between (I) and (II) domains, is a weak function of cosolute nature. Within the framework of this model, the solvent thermodynamic excesses in the inner and outer local domains are *ν*_W_^(I)^ = $N_{W}^{\left(I\right)}$(1− *K*^(I)^) and *ν*_W_^(II)^ = $N_{W}^{\left(I\right)}$(1 − *K*^(I)^), respectively, with *ν*_W_^(I)^ = $N_{W}^{\left(I\right)}$ and *ν*_W_^(II)^ = $N_{W}^{\left(I\right)}$ corresponding to the full depletion of cosolute in the two domains. The preferential-interaction coefficient considers cosolute–solvent partitioning in both domains. Consistent with Equation (27), we have the following:

(41) $$\gamma =\;\left(\nu_{W}^{\left(I\right)}+\nu_{W}^{\left(\mathrm{II}\right)}\right)\frac{C_{S}}{C_{W}}$$

Although *ν*_W_^(I)^ and *ν*_W_^(II)^ may significantly depend on cosolute nature, the ratio, *ν*_W_^(II)/^*ν*_W_^(I)^, which describes the relative change in interaction strength between (I) and (II) domains may be a weak function of cosolute nature.

Now that we have incorporated splitting of the local domain in the expression of the preferential-interaction coefficient, we can turn our attention to particle diffusiophoresis. It is expected that the actual particle [30] undergoing diffusion, {P}, consists of the bare particle, P, and inner local domain, with $N_{S}^{\left(I\right)}$ and $N_{W}^{\left(I\right)}$ cosolute (S) and solvent (W) molecules, respectively. We then assume that the transport of {P} is described by a simple diffusion equation [108]:

(42) $$v_{P}=-D_{P}\frac{\nabla \mu_{\left\{P\right\}}}{RT}$$

where *μ*_{P}_ is the chemical potential of {P}. Note that it is assumed that no explicit dependence of *v*_P_ on ▽*μ*_S_ is considered in Equation (42). Based on {P} composition, we express ▽*μ*_{P}_ in the following way:

(43) $$\nabla \mu_{\left\{P\right\}}=\;\nabla \mu_{P}+N_{W}^{\left(I\right)}\nabla \mu_{W}+N_{S}^{\left(I\right)}\nabla \mu_{S}$$

In other words, ▽*μ*_{P}_ will depend on ▽*μ*_W_ and ▽*μ*_S_ because solvent and cosolute molecules are present in the inner local domain. Since ▽*μ*_W_ and ▽*μ*_S_ are linked by the Gibbs–Duhem equation, *C*_S_▽*μ*_S_ + *C*_W_▽*μ*_W_ = 0, we can rewrite Equation (42) in the following way:

(44) $$v_{P}=-D_{P}\left(\frac{\nabla \mu_{P}}{RT}-\nu_{W}^{\left(I\right)}\frac{C_{S}}{C_{W}}\frac{\nabla \mu_{S}}{RT}\right)$$

where we have also used *ν*_W_^(I)^ = $N_{W}^{\left(I\right)}$(1 − *K*^(I)^) with *K*^(I)^ = ($N_{S}^{\left(I\right)}$/$N_{W}^{\left(I\right)}$)/(*C*_S_/*C*_W_). Finally, a comparison with Equation (14) shows that the hydrodynamic coefficient is as follows:

(45) $$\lambda =\nu_{W}^{\left(I\right)}\frac{C_{S}}{C_{W}}$$

This model correctly predicts that *λ* is proportional to *C*_S_. Furthermore, it is positive when there is an excess of solvent molecules binding to the particle, consistent with preferential hydration. On the other hand, *λ* is negative when cosolute binds to the particle. Within the framework of this model, *λ*/*γ* and ${\hat{D}}_{\mathrm{PS}}$/*γ* are essentially constants representing the inner and outer domains, respectively. Specifically, they are two complementary fractions of water thermodynamic excess:

(46a) $$\frac{\lambda }{\gamma }=\frac{\nu_{W}^{\left(I\right)}}{\nu_{W}^{\left(I\right)}+\nu_{W}^{\left(\mathrm{II}\right)}}$$

(46b) $$\frac{{\hat{D}}_{\mathrm{PS}}}{\gamma }=\frac{\nu_{W}^{\left(\mathrm{II}\right)}}{\nu_{W}^{\left(I\right)}+\nu_{W}^{\left(\mathrm{II}\right)}}$$

Experimental results on PEG in the preferential-hydration cases can be explained using this model. For example, *ν*_W_ = 1100 and ${\hat{D}}_{\mathrm{PS}}$/*γ* = 0.14 in the NaCl case corresponds to *ν*_W_^(I)^ = 945 and *ν*_W_^(II)^ = 155. The obtained values of ${\hat{D}}_{\mathrm{PS}}$/*γ* = 0.10–0.20 represent the fraction of solvent thermodynamic excess in the outer domain. If *ν*_W_^(II)/^*ν*_W_^(I)^ is a weak function of cosolute type, then ${\hat{D}}_{\mathrm{PS}}$/*γ* for different cosolutes tend to be close to each other even if *ν*_W_^(II)^ + *ν*_W_^(I)^ vary significantly from cosolute to cosolute. This is consistent with experimental findings.

In the presence of cosolute preferential binding, experimental results show that *γ* < 0 and ${\hat{D}}_{\mathrm{PS}}$/*γ* < 0. As discussed for PEG, in the presence of thiocyanate salts, actual cosolute binding occurs. This leads to negative values of *ν*_W_^(I)^. If we then assume that *ν*_W_^(II)^ of the outer domain is also negative, then we obtain ${\hat{D}}_{\mathrm{PS}}$ < 0 and ${\hat{D}}_{\mathrm{PS}}$/*γ* > 0, in disagreement with experimental results. Indeed, experimental findings can be explained only by assuming that *ν*_W_^(II)^ is positive even for urea and thiocyanate salts. For example, *ν*_W_ = −520 and ${\hat{D}}_{\mathrm{PS}}$/*γ* = −0.10 in the urea case leads to *ν*_W_^(I)^ = −570 and *ν*_W_^(II)^ = +50. This interesting result may be explained by considering that overall PEG–urea interactions are the result of an actual PEG–urea binding process (inner domain, *ν*_W_^(I)^ < 0) and residual excluded-volume interactions, which are associated with the outer domain and are responsible for *ν*_W_^(II)^ > 0 [142].

## 10. The lysozyme–Salt–Water System

Salt-induced protein diffusiophoresis, ${\hat{D}}_{\mathrm{PS}}$, and salt osmotic diffusion, ${\hat{D}}_{\mathrm{SP}}$, have been reported for lysozyme at pH 4.5 and 25 °C as a function of NaCl and KCl concentration, *C*_S_ [66,71,95]. Lysozyme is a globular protein with a molecular weight of 14.3 kDa that is chemically stable and commercially available at high purity. Due to these properties, this protein has been extensively studied. It is widely recognized as a model protein in the fields of biophysics and structural biology [130,143,144,145]. Since the lysozyme isoelectric point is 11, this protein is normally found to be positively charged. Indeed, lysozyme structural charge is known to be +10 at pH 4.5 [71,145], with chloride anions acting as counterions when NaCl or KCl are employed as supporting electrolytes.

**Figure 14:**
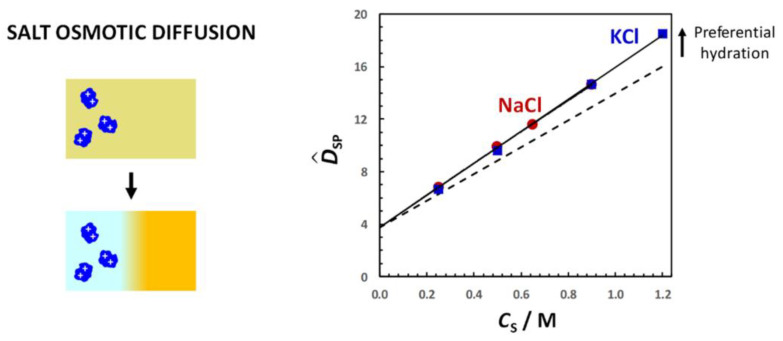
Osmotic diffusion of salt occurs from high to low lysozyme concentrations. Salt osmotic diffusion coefficient, ${\hat{D}}_{\mathrm{SP}}$, as a function of salt concentration, *C*_S_, in the NaCl (●) and KCl (■) cases [66,71]. Dashed line has same intercept as ${\hat{D}}_{\mathrm{SP}}$ data and slope equal to ${\bar{V}}_{P}$.

The experimental behavior of salt osmotic diffusion, ${\hat{D}}_{\mathrm{SP}}$(*C*_S_), is shown in Figure 14. Here, we can see that ${\hat{D}}_{\mathrm{SP}}$ linearly increases with *C*_S_. As in the case of PEG (see Figure 11), changing NaCl with KCl has essentially no appreciable effect on ${\hat{D}}_{\mathrm{SP}}$. However, ${\hat{D}}_{\mathrm{SP}}$ extrapolated at *C*_S_ = 0 is not zero but remains positive. This residual intercept is attributed to protein charge. Specifically, we have ${\hat{D}}_{\mathrm{SP}}$ ≈ |*Z*_P_|/2 + (${\bar{V}}_{P}$ + *ν*_W_${\bar{V}}_{W}$)*C*_S_ from ${\hat{D}}_{\mathrm{SP}}$ ≈ *C*_SP_ and Equation (29).

In Figure 14, all ${\hat{D}}_{\mathrm{SP}}$(*C*_S_) data are above the baseline |*Z*_P_|/2 + ${\bar{V}}_{P}$*C*_S_, thereby implying that lysozyme preferential hydration occurs in the presence of both salts. Application of Equations (18), (20) and (23) to ${\hat{D}}_{\mathrm{PS}}$(*C*_S_) and ${\hat{D}}_{\mathrm{SP}}$(*C*_S_) data, which confirms that ${\hat{D}}_{\mathrm{SP}}$≈*C*_SP_ is a suitable approximation, rigorously yields *γ*(*C*_S_). For both salt cases, the values of *Z*_P_ = 7.5 and *ν*_W_ = 150 are extracted. This charge value is somewhat lower than the protein structural charge of *Z*_P_ = 10 [145]. The observed difference is consistent with the presence of a Stern layer surrounding the charged protein with about three counterions bound to this globular macroion. Thus, the value of *Z*_P_ = 7.5 should be taken as an effective, renormalized charge, incorporating the contribution of the Stern layer [71].

**Figure 15:**
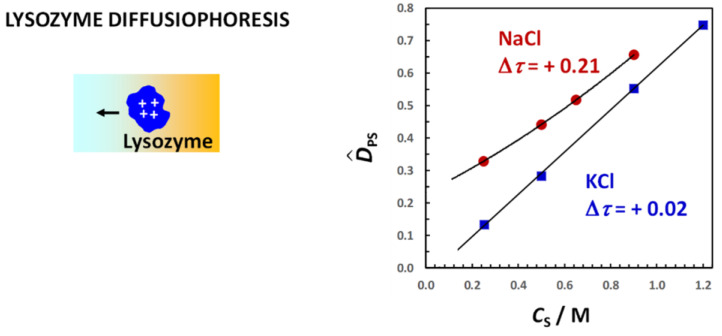
Diffusiophoresis of lysozyme occurs from high to low salt concentration. Lysozyme diffusiophoresis coefficient, ${\hat{D}}_{\mathrm{PS}}$, as a function of salt concentration, *C*_S_, in the NaCl (●) and KCl (■) cases [95]. Difference in counterion (Cl^−^) and coion (Na^+^ or K^+^) transference number, Δ*τ*, is reported for both salts.

The experimental behavior of lysozyme diffusiophoresis coefficient, ${\hat{D}}_{\mathrm{PS}}$(*C*_S_), is shown in Figure 15 for both salt cases [95]. Contrary to salt osmotic diffusion, there is a marked difference between the two ${\hat{D}}_{\mathrm{PS}}$(*C*_S_) curves. In the KCl case, ${\hat{D}}_{\mathrm{PS}}$ linearly increases with *C*_S_, with a small negative intercept, which will be further discussed later. This behavior is approximately the same as that shown for PEG with the same salt (see Figure 11). In contrast, ${\hat{D}}_{\mathrm{PS}}$ in the NaCl case is relatively large, with an evident positive intercept.

The difference between the two salt cases can be explained by considering Nernst–Planck equations. According to Equation (36), ${\hat{D}}_{\mathrm{PS}}$ is directly proportional to the difference between counterion (Cl^−^) and coion (Na^+^ or K^+^) transference numbers, Δ*τ*. Since Δ*τ* = 0.21 for NaCl and Δ*τ* = 0.02 for KCl [104,105,118], the diffusion potential (internal electric field) produced by salt gradient is significant only in the NaCl case. Thus, the difference between the two ${\hat{D}}_{\mathrm{PS}}$(*C*_S_) curves in Figure 15 is essentially attributed to the positive contribution of electrophoretic diffusiophoresis in the NaCl case.

## 11. Electrophoretic and Residual Diffusiophoresis

**Figure 16:**
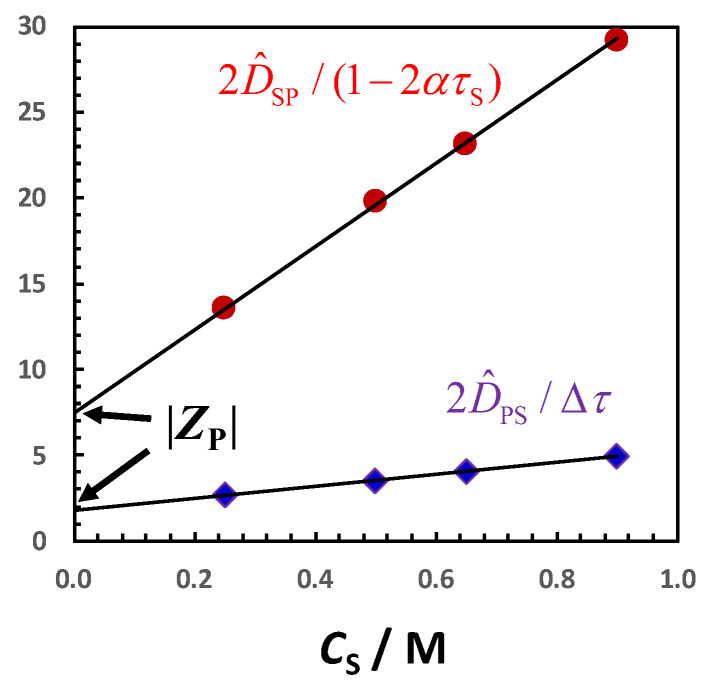
Plots of 2${\hat{D}}_{\mathrm{PS}}$/Δ*τ* (◆) and 2${\hat{D}}_{\mathrm{SP}}$/(1 − 2*ατ*_S_) (●) as a function of salt concentration, *C*_S_, in the NaCl case. According to Equations (36) and (38), both intercept values represent |*Z*_P_|.

For lysozymes with NaCl, it is possible to determine *Z*_P_ by extrapolating 2${\hat{D}}_{\mathrm{PS}}$/Δ*τ* to *C*_S_ = 0 according to Equation (36). In Figure 16, 2${\hat{D}}_{\mathrm{PS}}$/Δ*τ* is plotted as a function of *C*_S_. In the same figure, the plot of 2${\hat{D}}_{\mathrm{SP}}$/(1 − 2α*τ*_S_)≈2*C*_SP_, which also becomes *Z*_P_ at *C*_S_ = 0 according to Equation (38), is included for comparison. This latter plot yields *Z*_P_ = 7.5 at *C*_S_ = 0, as already discussed in Section 10. Interestingly, extrapolation of 2${\hat{D}}_{\mathrm{PS}}$/Δ*τ* yields a value of *Z*_P_ = 1.8, in substantial disagreement with osmotic diffusion data [71]. It will be now shown that this apparently low charge is mostly related to the electrical-double-layer effect, which is predicted to give rise to a significant electrophoretic effect even at salt concentrations of the order of 1–10 mM. In other words, the value of ${\hat{D}}_{\mathrm{PS}}$ significantly deviates from Equation (36) even at salt concentrations as low as *C*_S_ = 1 mM.

The effect of diffusion potential on diffusiophoresis of charged particles at low salt concentration can be described by applying the type of models employed in electrophoresis [17,146]. According to the electrical-double-layer (EDL) theory [147,148], the electrophoretic mobility of a particle is expected to drop dramatically as solution ionic strength increases. This effect is related to the diffuse layer surrounding charged particles in electrolyte solutions. Since an electric field tends to move the ion cloud in the opposite direction, a viscous drag applies to the macroion, thereby slowing down its motion. This electrophoretic effect can be incorporated into Equation (36) by writing the following:

(47) $${\hat{D}}_{\mathrm{PS}}=\sigma \frac{\left|Z_{P}\right|}{2}\Delta \tau$$

where *s* is an electrokinetic coefficient, with *σ*→1 in the limit of *C*_S_ = 0, consistent with Equation (36) [71]. Note that *Z*_P_ in Equation (47) represents the charge of the particle at its slip boundary. It can be approximately assumed to be the same as *Z*_P_ = 7.5 extracted from salt osmotic diffusion data because it takes into account the contribution of small ions located within the hydrodynamic volume of the diffusion particle. As *C*_S_ increases, *s* sharply decreases, making *s* |*Z*_P_| become a small fraction of |*Z*_P_| at salt concentrations of the order of 0.1 M [71].

**Figure 17:**
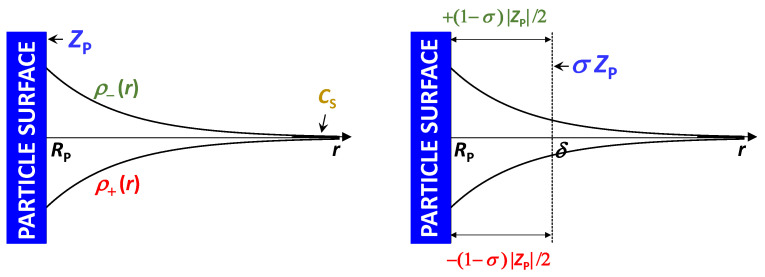
Coion (+) and counterion (−) density distributions (left), ρ_+_(*r*) and ρ_−_(*r*), as a function of the radial distance, *r*, starting from *r* = *R*_P_, the location of the slip surface of the spherical particle with positive charge, *Z*_P_. The common-ion effect is due to *Z*_P_ counterions. As *r* increases, ρ_+_ and ρ_−_ both approach the value of the bulk salt concentration, ρ_+_(∞) = ρ_−_(∞) = *C*_S_. Density distributions are assumed to respect symmetry conditions applicable to weakly charged globular macromolecules: *C*_S_ − ρ_+_(*r*) =ρ_−_(*r*) − *C*_S_. The radial distance, *r* = *δ*, delineates the boundary of the redefined macroion, with net charge, σ*Z*_P_ (right). The fluid domain enclosed by this boundary contains an excess of (1 − σ)*Z*_P_/2 counterions and a depletion of (1-σ)*Z*_P_/2 coions. The common-ion effect is then described by *Z*_P_ − [(1 − σ)*Z*_P_/2] = (1 + σ)*Z*_P_/2 counterions and 0 − [−(1 − σ)*Z*_P_/2] = (1 − σ)*Z*_P_/2, with the total number of common ions being still *Z*_P_.

It is important to observe that the introduction of *σ* in the Nernst–Planck equation for ${\hat{D}}_{\mathrm{PS}}$ does not require a modification of the Donnan expression of *C*_SP_= |*Z*_P_|/2 as long as the electrical potential on the macroion surface at the slip boundary, which is known as zeta potential, *ζ*, is weak [71]. This approximation is suitable for proteins because they have relatively low charge density on their surface. The presence of the electrophoretic effect may still be formally described using Nernst–Planck equations. However, the neutral protein component of the ternary mixture needs to be redefined. In its original description, the protein component consists of a macroion with charge, *Z*_P_, and |*Z*_P_| counterions. These counterions also act as common ions because they are shared with the salt component. To incorporate *σ*, the macroion needs to be redefined so that it has an effective charge, *σZ*_P_, consistent with Equation (47). The newly defined macroion must formally contain the macroion itself (with charge *Z*_P_) and a contiguous fraction of the diffuse layer, such that the net macroion charge reduces from *Z*_P_ to *σZ*_P_. In other words, the charge of the inner fraction of the diffuse layer must be −(1 − *σ*)*Z*_P_ [71]. As further described in Figure 17, the inner fraction of the diffuse layer contains an excess of counterions, (1 − *σ*)|*Z*_P_|/2, and a corresponding depletion of coions, −(1 − *σ*)|*Z*_P_|/2. Thus, the redefined macroion is formally composed by the original macroion to which (1 − *σ*)|*Z*_P_|/2 counterions are added and (1 − *σ*)|*Z*_P_|/2 coions are removed. The common ions stoichiometrically balancing this macroion must consist of (1 + *σ*)|*Z*_P_|/2 counterions and (1 − *σ*)|*Z*_P_|/2 coions. In other words, the common ion is redefined as a combination of counterions and coions. This combination ensures that the total number of common ions is still |*Z*_P_| and *C*_SP_ = |*Z*_P_|/2 remains valid.

The incorporation of *σ* in Equation (37) also has an impact on the expression of *λ* given by Equation (37). After inserting the expression of ${\hat{D}}_{\mathrm{PS}}$ given by Equation (47) in Equation (18) and setting *γ* = *C*_SP_ = |*Z*_P_|/2, we obtain the following [71]:

(48) $$\lambda =|Z_{P}|\;\frac{(1+\sigma )\tau_{S}+(1-\sigma )\tau_{C}}{2}$$

where (1 + *σ*)|*Z*_P_|*τ*_S_/2 and (1 − *σ*)|*Z*_P_|*τ*_C_/2 terms in Equation (48) replace |*Z*_P_|*τ*_S_ in Equation (37) as a direct consequence of the redefinition of the protein component.

The electrophoretic effect is described by the function *σ*(*C*_S_) starting from *σ* = 1 at *C*_S_ = 0. As *C*_S_ increases, *σ* sharply decreases, making *σZ*_P_ become just a small fraction of *Z*_P_. To appreciate the significance of this electrokinetic factor for proteins and globular colloidal particles in general, the following expression may be considered [71]:

(49) $$\sigma =\frac{f(\kappa R_{P})}{1+\kappa R_{P}}$$

where *R*_P_ is the particle hydrodynamic radius (radius at the slip boundary), *κ* ≡ (8000*pN_A_λ_B_I*)^1/2^ is the Debye constant, with *I* being the salt ionic strength, *N_A_* the Avogadro’s number and *λ_B_* the Bjerrum length (0.7151 nm for water at 25 °C), and *f*(*κR*_P_) is a corrective function. In Equation (49), 1/(1 + *κR*_P_) is the dominant factor describing the electrophoretic effect. This factor also describes the effect of ionic strength on zeta potential, *ζ*, according to F*ζ*/R*T* = *Z*_P_(*λ_B_*/*R*_P_)/(1 + *κR*_P_).

One of the most employed expression of *f*(*κR*_P_) is Henry’s function [17]:

(50) $$f(x)=\frac{3}{2}-e^{x}\left[\frac{15}{2}E_{7}(x)-3E_{5}(x)\right]$$

where *E_n_*(*x*) ≡ $\int_{1}^{\infty }$*t*^−*n*^*e*^−*xt*^*dt*. This takes into accounts the curvature of spherical particles with *f*(0) = 1 (Debye–Huckel limit) and *f*(∞) = 3/2 for a planar surface (Smoluckowski limit). For lysozyme, the electrophoretic factor can be evaluated as a function of salt concentration at 25 °C using Equations (49) and (50) and lysozyme hydrodynamic radius, *R*_P_ = 1.863 nm. Related data are reported in Table 1.

Note that EDL theory is expected to be accurate at relatively low salt concentrations (≈0.1 M or less) due to ion–ion steric effects and related ion–ion correlation. Nonetheless, Table 1 allows us to appreciate that the most significant change in *s* occurs within the reliable low salt concentration range. The 2${\hat{D}}_{\mathrm{PS}}$/Δ*τ* data in Figure 16 refer to salt concentrations above 0.1 M. According to Table 1, we have *σ* ≈ 0.2 in this concentration range. Thus, the extrapolation of these data at *C*_S_ = 0 is predicted to yield a charge that is about 20% of the value extracted from 2${\hat{D}}_{\mathrm{SP}}$/(1 − 2α*τ*_S_) data. This is consistent with the discrepancy observed in Figure 16. Thus, the introduction of the electrophoretic factor in Equation (47) represents a critical correction to Nernst–Planck equations so that these equations can accurately describe the limiting behavior of experimental data obtained at salt concentrations of ≈0.1 M and higher.

**Figure 18:**
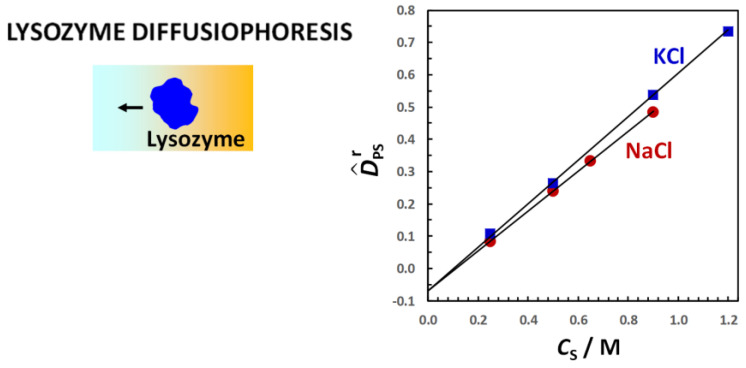
Diffusiophoresis of lysozyme occurs from high to low salt concentrations due to preferential hydration. Residual lysozyme diffusiophoresis coefficient, ${\hat{D}}_{\mathrm{PS}}^{\;r}$, as a function of salt concentration, *C*_S_, in the NaCl (●) and KCl (■) cases [95].

We are now in a position to quantitatively examine the experimental behavior of ${\hat{D}}_{\mathrm{PS}}$(*C*_S_) in Figure 15. It is convenient to write the following:

(51) $${\hat{D}}_{\mathrm{PS}}={\hat{D}}_{\mathrm{PS}}^{\;e}+{\hat{D}}_{\mathrm{PS}}^{\;r}$$

where ${\hat{D}}_{\mathrm{PS}}^{\;e}$ is the electrophoretic contribution to diffusiophoresis, which is evaluated using *Z*_P_ = 7.5 in Equation (47). The second term in Equation (51), ${\hat{D}}_{\mathrm{PS}}^{\;r}$, is a residual contribution, which is calculated from ${\hat{D}}_{\mathrm{PS}}$–${\hat{D}}_{\mathrm{PS}}^{\;e}$ [71,95]. The corresponding plots of ${\hat{D}}_{\mathrm{PS}}^{\;r}$(*C*_S_) are shown in Figure 18 for both salts. Here, we can see that ${\hat{D}}_{\mathrm{PS}}^{\;r}$ linearly increases with *C*_S_ for both salt cases. Moreover, changing NaCl with KCl now has a small effect on ${\hat{D}}_{\mathrm{PS}}$. This analysis confirms that the difference between the two salt cases shown in Figure 14 is due to electrophoretic diffusiophoresis. Interestingly, ${\hat{D}}_{\mathrm{PS}}^{\;r}$ retains a residual negative intercept, which is virtually the same for both salts, i.e., ${\hat{D}}_{\mathrm{PS}}^{\;r}$(0) = −0.07 ± 0.01. This result, which is not observed in the PEG case, hardly depends on the accuracy of ${\hat{D}}_{\mathrm{PS}}^{\;e}$ determination because its contribution is invariably very small in the KCl case. This negative intercept can be attributed [95] to another small EDL effect known as the chemiphoresis [15] mechanism. Specifically, a salt concentration gradient is responsible for a corresponding gradient of ionic strength within the EDL of a spherical particle, which causes particle diffusiophoresis from low to high salt concentrations. Chemiphoresis negatively contributes to diffusiophoresis and is predicted to be the same for both salts. Since it is typically small, it will be ignored in the analysis below.

The slope of ${\hat{D}}_{\mathrm{PS}}^{\;r}$(*C*_S_) in Figure 18 can be described within the framework of the preferential-hydration mechanism and the local-domain model already employed for PEG. As in the diffusiophoresis coefficient, we split the preferential-interaction coefficient, *g*, into two terms:

(52) $$\gamma =\gamma^{e}+\gamma^{r}$$

where *γ*^e^ = |*Z*_P_|/2 and *γ*^r^ = *ν*_W_$\bar{V}$_W_*C*_S_. We can then determine the ratio, ${\hat{D}}_{\mathrm{PS}}^{\;r}$/*γ*^r^, for both salt cases. The obtained values of ${\hat{D}}_{\mathrm{PS}}^{\;r}$/*γ*^r^ = 0.23 (NaCl) and ${\hat{D}}_{\mathrm{PS}}^{\;r}$/*γ*^r^ = 0.25 (KCl) for lysozyme [108] are somewhat higher than those shown earlier for PEG. Nonetheless, they are still a small fraction of one, consistent with the local-domain model. If we use Equation (46b) with *ν*_W_ = 150 and ${\hat{D}}_{\mathrm{PS}}^{\;r}$/*γ*^r^ = 0.24 for both salts, we obtain *ν*_W_^(I)^ = 115 and *ν*_W_^(II)^ = 35.

## 12. Concluding Remarks

There is a growing number of experimental observations, applications and future opportunities related to diffusiophoresis [13]. This review has focused on the use of Rayleigh interferometry as a precise experimental technique for measuring multicomponent-diffusion coefficients on ternary aqueous mixtures of macromolecules (PEG or lysozyme) in the presence of cosolutes (salts or osmolytes). These data are employed to characterize the behavior of the diffusiophoresis coefficient, ${\hat{D}}_{\mathrm{PS}}$, as a function of cosolute concentration, *C*_S_. These data are also used to determine the corresponding cosolute osmotic diffusion coefficient, ${\hat{D}}_{\mathrm{SP}}$. Examination of diffusiophoresis within the framework of non-equilibrium thermodynamics allows us to relate ${\hat{D}}_{\mathrm{PS}}$ = *γ* − *λ* to the preferential-interaction coefficient, *γ*, and the Onsager transport coefficient, *λ*. The combination of ${\hat{D}}_{\mathrm{PS}}$ and ${\hat{D}}_{\mathrm{SP}}$ data enables the characterization of *γ* and *λ*. PEG diffusiophoresis in response to salt or osmolyte gradients is driven by a preferential-hydration mechanism, while lysozyme diffusiophoresis in response to salt gradients is driven by both preferential-hydration and electrophoretic mechanisms, the latter being the mechanism extensively described in the diffusiophoresis literature [12,13,19,20]. While the same effective charge (*Z*_P_ = 7.5) successfully explains both ${\hat{D}}_{\mathrm{PS}}$ and ${\hat{D}}_{\mathrm{SP}}$ data for lysozyme, it is not clear whether this agreement remains valid for other charged particles. Since proteins are weakly charged macromolecules, future multicomponent-diffusion studies should compare salt-induced diffusiophoresis with salt osmotic diffusion for strongly charged macromolecules such as nucleic acids, micelles and vesicles. It would also be interesting to explore diffusiophoresis for charged macromolecules in which the electrophoretic mechanism drives diffusiophoresis from low to high salt concentration while the preferential-hydration mechanism drives diffusiophoresis in the opposite direction. In this case, one can predict that there is a salt concentration at which the sign of ${\hat{D}}_{\mathrm{PS}}$ changes from negative to positive. This implies that a steady salt concentration gradient would focus macromolecules at a specific location, corresponding to the salt concentration where ${\hat{D}}_{\mathrm{PS}}$ = 0. This situation is analogous to isoelectric focusing in electrophoresis. It is also important to examine diffusiophoresis for neutral globular nanoparticles possessing interfacial PEG moieties. This is important for assessing the extent to which the values of preferential-hydration parameters, *ν*_W_ (per ethoxy group) and ${\hat{D}}_{\mathrm{PS}}$/*γ* extracted for PEG, are applicable to PEG-based nanoparticles. Finally, future studies should also systematically investigate diffusiophoresis in the proximity of liquid–liquid phase separation (or cloud point). It is known that proteins [149,150], PEG [151] and micellar systems [152] are susceptible to phase separation in the presence of certain salts. Recent studies on tyloxapol micelles [23] indicate that salt-induced diffusiophoresis of colloidal particles dominates over their Brownian motion near phase-separation conditions due to a large osmotic compressibility. These studies would be useful for understanding the diffusiophoresis of nanoparticles in the presence of salt brines, with potential applications of diffusiophoresis to enhanced oil recovery [35].

## Figures and Tables

**Table 1:** Electrokinetic factor, *σ*, as a function of salt concentration, *C*_S_.

*C*_S_/M	*κR*_P_	*f*(*κR*_P_)	*σ*	*C*_S_/M	*κR*_P_	*f*(*κR*_P_)	*σ*
0.001	0.194	1.002	0.8392	0.30	3.357	1.113	0.2554
0.002	0.274	1.006	0.7895	0.40	3.876	1.129	0.2315
0.005	0.433	1.010	0.7045	0.50	4.334	1.142	0.2141
0.010	0.613	1.013	0.6280	0.60	4.747	1.153	0.2006
0.020	0.867	1.022	0.5476	0.70	5.128	1.163	0.1898
0.050	1.370	1.039	0.4385	0.80	5.482	1.171	0.1807
0.10	1.938	1.063	0.3617	0.90	5.814	1.179	0.1730
0.20	2.741	1.092	0.2919	1.00	6.129	1.186	0.1664
